# An apple sucrose transporter MdSUT2.2 is a phosphorylation target for protein kinase MdCIPK22 in response to drought

**DOI:** 10.1111/pbi.13003

**Published:** 2018-10-02

**Authors:** Qi‐Jun Ma, Mei‐Hong Sun, Jing Lu, Hui Kang, Chun‐Xiang You, Yu‐Jin Hao

**Affiliations:** ^1^ National Key Laboratory of Crop Biology MOA Key Laboratory of Horticultural Crop Biology and Germplasm Innovation in Huanghuai Region College of Horticulture Science and Engineering Shandong Agricultural University Tai‐An Shandong China

**Keywords:** drought, sugar, MdSUT2.2, MdCIPK22, apple

## Abstract

Sugars increase with drought stress in plants and accumulate in the vacuole. However, the exact molecular mechanism underlying this process is not clear yet. In this study, protein interaction and phosphorylation experiments were conducted for sucrose transporter and CIPK kinase in apple. The specific phosphorylation site of sucrose transporter was identified with mass spectrometry. Transgenic analyses were performed to characterize their biological function. It was found that overexpression of sucrose transporter gene *MdSUT2.2* in apple plants promoted sugar accumulation and drought tolerance. MdSUT2.2 protein was phosphorylated at Ser^381^ site in response to drought. A DUALmembrane system using MdSUT2.2 as bait through an apple cDNA library got a protein kinase MdCIPK22. Bimolecular fluorescence complementary (BiFC), pull‐down and co‐immunoprecipitation (Co‐IP) assays further demonstrated that MdCIPK22 interacted with MdSUT2.2. A series of transgenic analysis showed that MdCIPK22 was required for the drought‐induced phosphylation at Ser^381^ site of MdSUT2.2 protein, and that it enhanced the stability and transport activity of MdSUT2.2 protein. Finally, it was found that *MdCIPK22* overexpression promoted sugar accumulation and improved drought tolerance in an MdSUT2.2‐dependent manner in transgenic apple plants. MdCIPK22‐MdSUT2.2 regulatory module shed light on the molecular mechanism by which plant accumulates sugars and enhances tolerance in response to drought stress.

## Background

Drought is a meteorological term and is commonly defined as a period without significant rainfall. Generally drought stress occurs when the available water in the soil is reduced and atmospheric conditions cause continuous loss of water by transpiration or evaporation (Jaleel *et al*., 2009). Terminal drought conditions bring about a progressive decrease in soil water availability to plants and cause premature plant death, while intermittent drought conditions affect the plant growth and development.

A large number of physiological, biochemical and molecular processes at the cellular or whole plant level are altered in response to drought and play an important role in mitigating stress (Athar and Ashraf, 2009). Osmotic adjustment is one of the important physiological methods to cope with drought stress in plants. Synthesis of compatible solutes such as carbohydrates prevents water loss from cells and plays an important role in turgor maintenance under stresses (Bhargava and Sawant, 2013; Parvaiz and Satyawati, 2008). Sugars have emerged as important components of osmotic potential in response to abiotic stresses. And in turn abiotic stresses play a central role in carbohydrate accumulation and affects soluble sugar content in growing plants (Kempa *et al*., 2008). At the molecular level, a series of genes are induced by stresses and involved in the stress‐induced sugar accumulation (Chinnusamy *et al*., 2004; Shinozaki and Yamaguchi‐Shinozaki, 2007). Abscisic acid (ABA) is an integral regulator in response to drought stress as well as in regulation of stress‐induced metabolic adjustments (Cutler *et al*., 2010; Hubbard *et al*., 2010; Kempa *et al*., 2008; Raghavendra *et al*., 2010; Umezawa *et al*., 2010). Several key transcription factors, such as ABA‐responsive element‐binding protein 1 (AREB1)/ABA‐responsive element (ABRE)‐binding factor 2 (ABF2) and dehydration‐responsive element (dre)‐binding protein 2A (DREB2A), have been identified to involve in drought stress signal transduction. Overexpressing DREB1A/C‐repeat binding factor 3 (CBF3) results in an increased accumulation of soluble sugars (glucose, fructose, sucrose and raffinose), thereby improving drought and cold tolerance in transgenic plants (Cook *et al*., 2004; Maruyama *et al*., 2009).

The sugar transporters SUTs or SWEETS function not only in the loading of sucrose into the phloem and the sink tissues, but also in the accumulation of soluble sugars in cells (Reinders *et al*., 2006). Seventeen members of SWEETs fall into four phylogenetic clades, i.e. SWEET1 to SWEET3 in clade I, SWEET4 to SWEET8 in clade II, SWEET 9 to SWEET15 in Clade III, and SWEET16 to SWEET17 in clade IV (Eom *et al*., 2015). Meanwhile plant SUTs are divided into five major clades, i.e. SUT1 to SUT5 (Kühn *et al*., 2003). Members of SUT1 and SUT3 clades are localized at the plasma membrane and are responsible for phloem loading or sucrose import into sink tissues (Slewinski *et al*., 2009). SUT2 functions not only as a sucrose transporter in the plasma membrane, but also as a low‐affinity sugar sensor by measuring the sugar transport rate and regulating plant development (Schulze *et al*., 2000). For example, *Arabidopsis* AtSUT2 and *Plantago major* PmSUT2 are predicted to be phloem‐localized and to catalyze sucrose loading into the sieve element‐companion cell complex (Kühn *et al*., 2003). In addition, some members of SUT4 clade such as *Arabidopsis* AtSUT4, barley HvSUT2, rice OsSUT2, wheat TaSUT2, *Lotus japonicus* LjSUT4, and poplar PtaSUT4 are localized in vacuolar membranes and function as sucrose/H^+^ symporter (Endler *et al*., 2006; Frost *et al*., 2012; Reinders *et al*., 2006). And it has been suggested that HvSUT2, LjSUC4, and AtSUC4 might be responsible for the release of sucrose from the vacuole (Reinders *et al*., 2006; Schulze *et al*., 2000).

In various plant species, the expression of *SUTs* is regulated by various abiotic stresses. Citrus *CiSUT1* and *CiSUT2* are essential for soluble sugar accumulation in response to cold stresses (Wei *et al*., 2016). The expression of *SUC1* and *SUC2* are up‐regulated by low temperature in *Arabidopsis* (Lundmark *et al*., 2006). In rice, *SUT1* and *SUT2* are involved in salt and drought stress responses (Ibraheem *et al*., 2011; Siahpoosh *et al*., 2012). In celery, *AgSUT1* suppression reduces the sensitivity to salt stress (Gong *et al*., 2015). *PtaSUT4‐RNAi* increased vacuolar sequestration of sucrose in *Populus* plants under drought stress (Frost *et al*., 2012).

Besides transcriptional regulation, the transport activity of sugar transporter is affected by posttranslational modification. It is reported that protein phosphorylation plays a key role in mediating sucrose regulation of *BvSUT1* transcription and sucrose transport activity (Ransom‐Hodgkins *et al*., 2003). Low temperature induces the phosphorylation of TMT‐type monosaccharide carriers at amino acid positions S385 in TMT1 and S376 in TMT2 (Schulze *et al*., 2000). SUTs proteins are also predicted to be the target for phosphorylation by serine/threonine kinases (Nühse *et al*., 2004; Roblin *et al*., 1998). However, the exact phosphorylation sites and responsible kinases for SUT phosphorylation remain unclear yet.

Abiotic stresses including drought often elicit an increase in free Ca^2+^ concentration in cells, which can be accepted as a secondary messenger to transduce the cellular responses. This process is always associated with the calcium signalling, which is mediated by four kinds of calcium sensors: calcium‐dependent protein kinases (CDPKs), calmodulin (CaM), calmodulin‐like proteins (CML) and calcineurin B‐like (CBL) protein (Huang *et al*., 2011). Among these calcium sensors, CBL interacts with and regulates the CBL‐interacting protein kinases (CIPKs) in plant (Cheong *et al*., 2003). It has been well documented that CBL‐CIPK network is involved in the response to stress, and transduces the stress signal to the nucleus to regulate the expression of related genes (Luan, 2009; Tuteja and Sopory, 2008). A physiological function has been established for a few CIPK proteins in response to abiotic stress in various plant species (Chen *et al*., 2012; Mahajan *et al*., 2006; Pandey *et al*., 2008). CIPK23 functions as a major component of ABA‐regulated drought tolerance in *Arabidopsis*. It is recruited by and interacted with two calcium sensors (CBL1 and CBL9) to the plasma membrane to regulate the potassium channel AKT1 (Cheong *et al*., 2007; Li *et al*., 2006; Xu *et al*., 2006). However, it is largely unknown concerning if and how CIPKs regulate sugar accumulation in response to stresses.

In apple, it is found that the MdAREB2‐MdSUT2.2 (formly named MdSUT2) regulon plays an important role in ABA‐induced sugar accumulation in apple (Ma *et al*., 2017a), and that a protein kinase MdCIPK22 interacts with and phosphorylates MdABRE2 to regulate its transcription activity in response to ABA signal (Ma *et al*., 2017b). This study provides evidence for MdCIPK22 as a positive regulator of the sucrose transporter MdSUT2.2. The roles of MdCIPK22 and MdSUT2.2 in stress‐induced sugar accumulation was elucidated and discussed.

## Results

### Overexpression of *MdSUT2.2* increases sugar content and drought tolerance

Phylogenetic tree demonstrated that two genes *MDP0000850943* (*MdSUT2.1*) and *MDP0000277235* (*MdSUT2.2*, formly named *MdSUT2*) in Genome Database for Rosaceae (https://www.rosaceae.org/) are closely homology with a sucrose transporter gene *AtSUT2* (Figure S1). The similarity analysis using DNAMAN software shows that the amino acid sequence similarity between AtSUT2 and MdSUT2.1 is 69.40%, while that between AtSUT2 and MdSUT2.2 is 65.95% (Figure S2). In addition, it was found that the expression levels of *MdSUT2.1* and *MdSUT2.2* genes increased with drought treatment (Figure S3).

**Figure 1:**
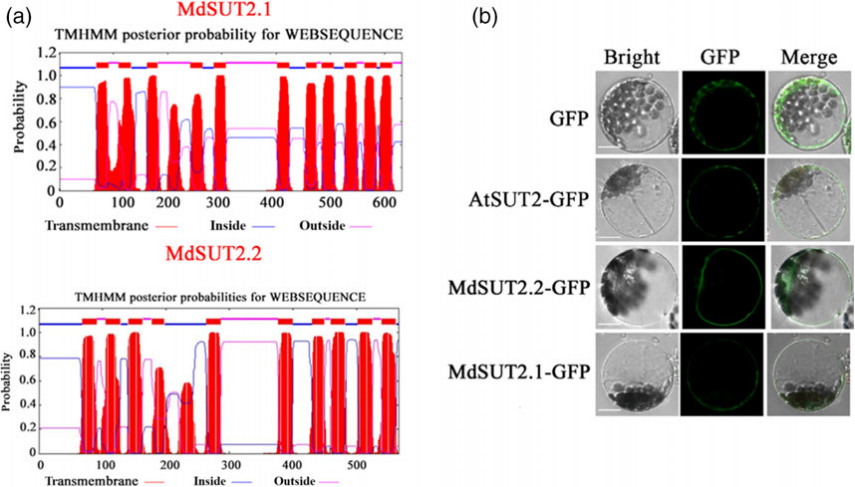
The transmembrane and subcellular localization of MdSUT2.1, MdSUT2.2. (a) Prediction of transmembrane analysis of MdSUT2.1 and MdSUT2.2. (b) The subcellular localization of MdSUT2.1, MdSUT2.2 and AtSUT2. Scale bar 100 μm.

**Figure 2:**
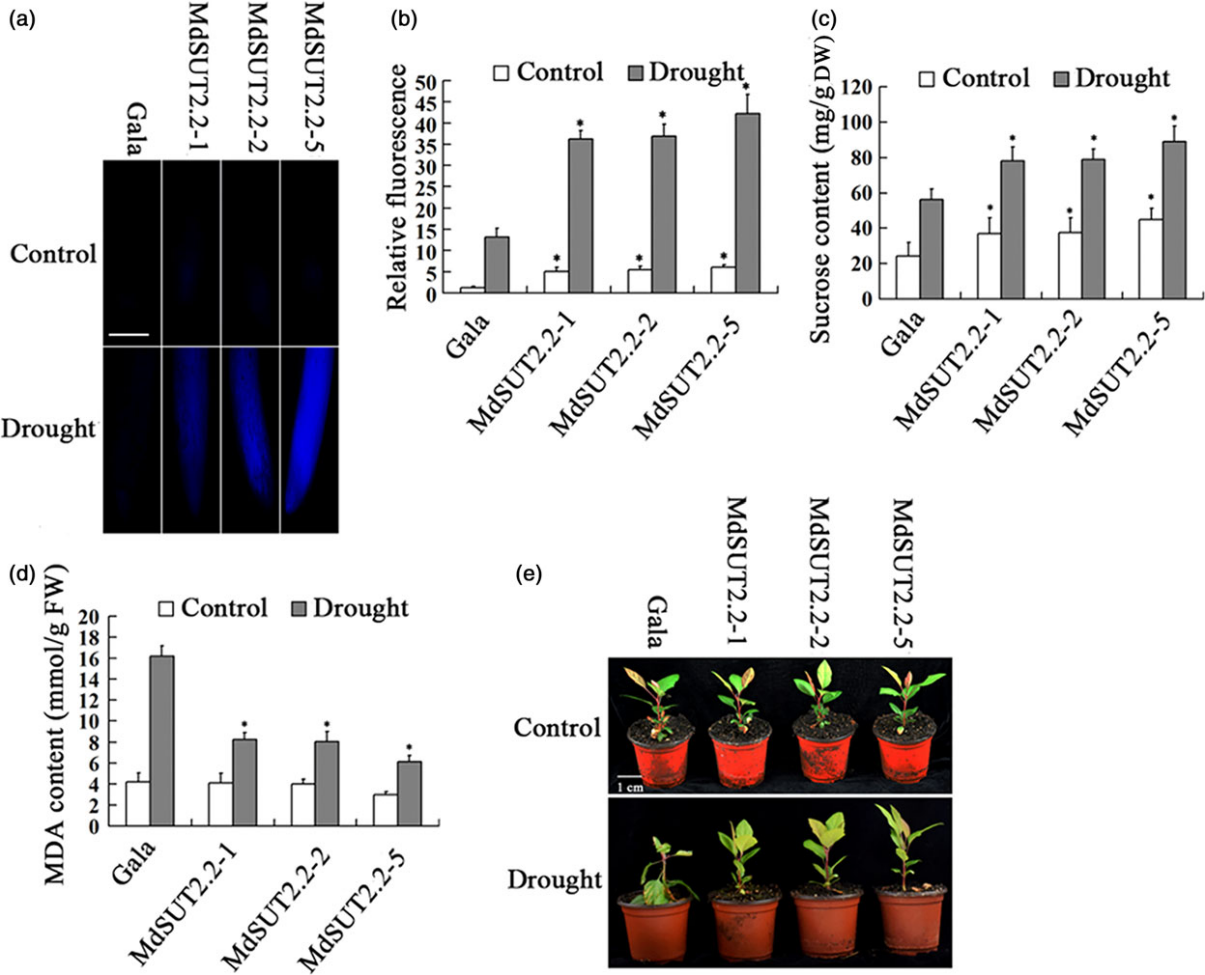
*MdSUT2.2* overexpression increased drought tolerance in transgenic apple plant. (a) Esculin uptake assay of sucrose transport activity in roots of three transgenic lines and the WT control treated with or without drought; (b) Relative fluorescence intensity in (a); (c) Sucrose contents in dry weight of three transgenic lines and Gala with or without drought. (d) MDA content in of fresh weight transgenic and the WT control plants treated with or without drought. (e) Tolerance observation of transgenic and the WT control plants treated with or without drought. n.s., *P* > 0.01;*P < 0.01;**P < 0.001.

Prediction of transmembrane analysis showed that AtSUT2, MdSUT2.1 and MdSUT2.2 proteins are characterized by 12, 11 and 10 transmembrane domains respectively (Figures 1a and S4). Interestingly, the subcellular localizations using *Arabidopsis* protoplast showed that MdSUT2.2 is localized to the tonoplast, while AtSUT2 and MdSUT2.1 to the plasma membrane (Figure 1b). Therefore, MdSUT2.2 was chosen for further investigation. To further characterize the biological function of *MdSUT2.2* in apple *in planta*, overexpression vector 35S::MdSUT2.2‐Myc was constructed and transformed into apple cultivar ‘Gala’. Three transgenic lines, i.e. MdSUT2.2‐1, MdSUT2.2‐2 and MdSUT2.2‐5 (Ma *et al*., 2017a), were chosen for further investigation without or with drought. To monitor sucrose transporter activity in roots, a fluorescent sucrose analog esculin was used (Gora *et al*., 2012). Esculin uptake assay demonstrated that three transgenic lines exhibited stronger esculin fluorescence intensity in roots than the WT control under conditions without or with drought (Figure 2a,b), indicated that *MdSUT2.2* overexpression enhanced sucrose transport activity. Moreover, three transgenic lines accumulated more sucrose and soluble sugars (Figures 2c and S5). Finally, they exhibited higher tolerance as indicated by less Malondialdehyde (MDA) generation and more water content under drought stress than the WT control (Figures 2d,e and S5). These results indicated that *MdSUT2.2* overexpression promoted sugar accumulation and increased drought tolerance in apple.

### Drought induces phosphorylation of MdSUT2.2 protein at residue Ser381

**Figure 3:**
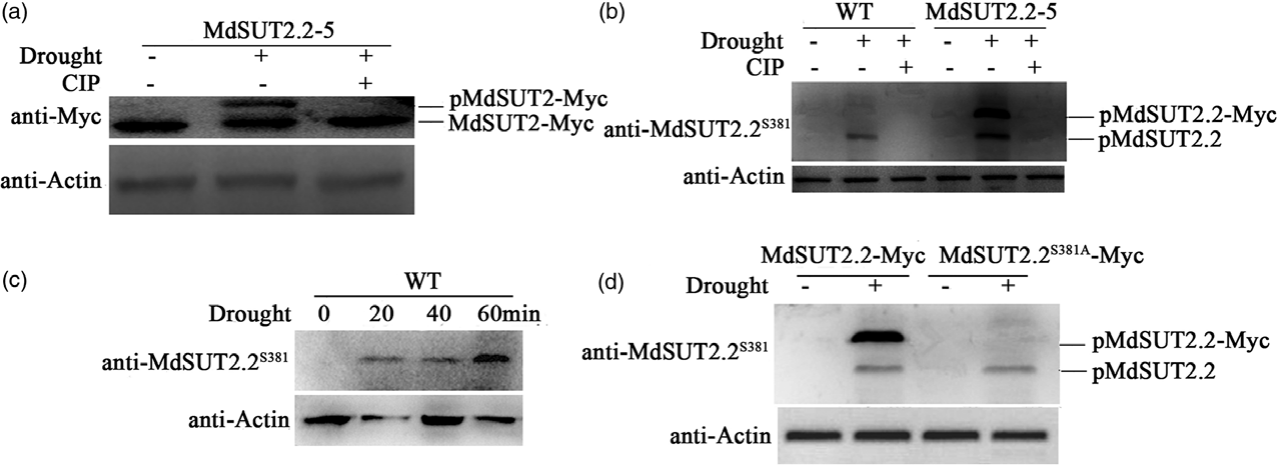
Drought induces phosphorylation for MdSUT2.2 protein (a) Gel‐shift assay of MdSUT2.2 protein in transgenic apple line MdSUT2.2‐5 using anti‐Myc antibody. Plants were treated with drought stress or together with CIP for 1 h; (b) Western blot detected the phosphorylation of the MdSUT2.2 protein in the WT and transgenic plant MdSUT2.2‐5 using a specific antibody anti‐MdSUT2.2^S381^ against the phosphorylation site at residue 381; (c) Drought‐induced phosphorylation of MdSUT2.2 protein increased with treatment time (min). WT ‘Gala’ apple plants were treated with different time (0, 20, 40, or 60 min); (d) Drought (PEG‐mimic)‐induced phosphorylation of MdSUT2.2 protein disappeared in MdSUT^S381A^‐Myc transgenic apple calli using anti‐MdSUT2.2^S381^ antibody. For Western blot assays, actin was used as a loading control to ensure identity in the amounts of protein.

35S::MdSUT2.2‐Myc transgenic apple line MdSUT2.2‐5 was used to examine if MdSUT2.2 protein occurs posttranslational modification in response to drought. Total proteins were extracted from MdSUT2.2‐5 transgenic plants treated with or without drought stress, and then used for immunoblotting analysis with an anti‐Myc antibody. There is only one fast‐moving band observed in MdSUT2.2‐5 transgenic plants without drought treatment. Besides the fast‐moving band, however, an additional slow‐moving band appeared in transgenic plants treated with drought (Figure 3a), indicating that drought induced a possible posttranslational modification for MdSUT2.2 protein. Furthermore, treatment with calf intestine alkaline phosphatase (CIP) was conducted under drought conditions. It acts to cleave phosphate residues away from phospho‐peptides. As a result, the slow‐moving band disappeared in drought‐treated plants (Figure 3a), indicating that the drought‐induced posttranslational modification for MdSUT2.2 protein is phosphorylation.

To identify the possible phosphorylation sites in MdSUT2.2 protein, the slow‐moving proteins were collected with anti‐Myc antibody‐conjugated agarose beads and separated with SDS‐PAGE. After proteolytic digestion and purification, the protein sample was analysed with liquid chromatography‐tandem mass spectrometry (LC‐MS/MS). The result indicated that a residue serine at site 381 (Ser^381^) was a potential phosphorylation site in MdSUT2.2 proteins under drought treatment (Figure S6).

Subsequently, a phospho‐specific monoclonal antibody (S^381^) named as anti‐MdSUT2.2^S381^ was prepared to recognize against phosphorylation site at residue Ser^381^ of MdSUT2.2 protein. Specific anti‐MdSUT2.2^S381^ antibody was used for immnobloting analysis. The result showed that it recognized drought‐induced phosphorylation of MdSUT2.2 protein in the WT apple plant, as well as MdSUT2.2 and MdSUT2.2‐Myc proteins in MdSUT2.2‐5 transgenic apple plant, indicating that drought induced phosphorylation of MdSUT2.2 proteins at site Ser^381^ (Figure 3b). Moreover, apple plantlets were treated with drought for 0, 10, 20, 30 and 60 min respectively, to examine whether treatment time affects phosphorylation. The results showed that phosphorylation intensity of MdSUT2.2 proteins increased gradually with treatment duration (Figure 3c).

For examining the influence of Ser381 on phosphorylation in response to drought, apple calli of cultivar ‘Orin’ were used for genetic transformation, and PEG treatment was applied to mimic drought. Firstly, it was found in Western blotting with an anti‐MdSUT2.2^381^ antibody that PEG induced phosphorylation of MdSUT2.2 in apple calli, just as drought did in apple plants (Figure S7). To determine whether Ser^381^ is required for drought‐induced phosphorylation of MdSUT2.2 protein, a serine‐to‐alanine mutation to Ser^381^ site was made in MdSUT2.2. Then, the resultant vector 35S::MdSUT2.2^S381A^‐Myc and 35S::MdSUT2.2‐Myc was genetically transformed into apple calli of ‘Orin’ cultivar (Figure S8a). Transgenic calli was then used for immunoblot assay using anti‐MdSUT2.2^S381^ specific antibody. The result demonstrated that mutation from Ser^381^ to Ala^381^ completely abolished phosphorylation modification for MdSUT2.2‐Myc protein (Figure 3d), indicating a crucial role of Ser^381^ site for PEG (drought)‐induced phosphorylation of MdSUT2.2 protein.

In addition, it was found that 35S::MdSUT2.2^S381A^‐Myc transgenic calli accumulated less sugars and more MDA than 35S::MdSUT2.2‐Myc calli, but more sugars and less MDA than the WT control (Figure S8b,c,d). Taken together, these data indicated that Ser^381^ is a crucial site for both phosphorylation and biological function of MdSUT2.2 protein in response to drought or PEG stress.

### MdSUT2.2 interacts with MdCIPK22

**Figure 4:**
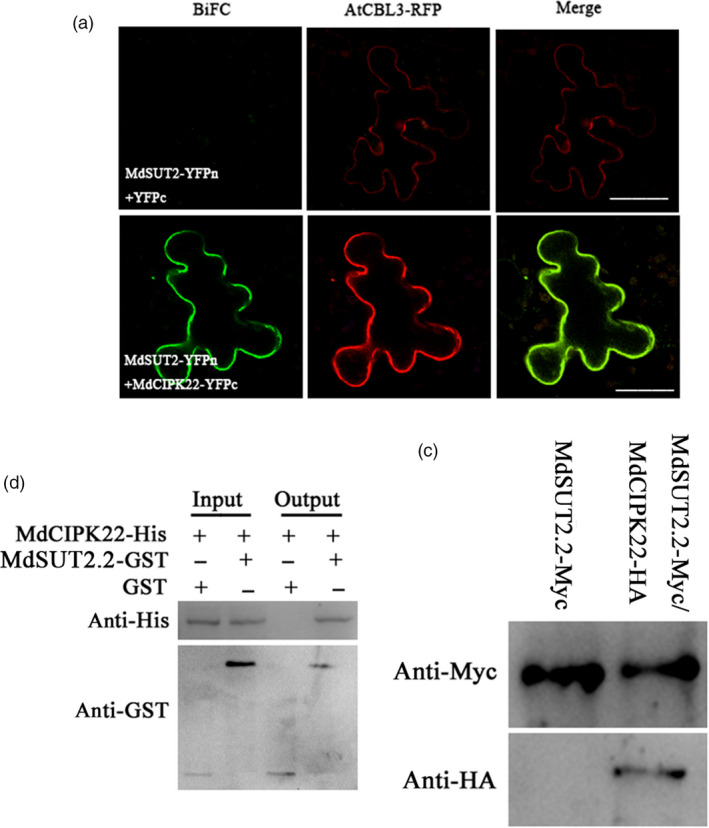
MdSUT2.2 interacts with MdCIPK22. (a) BiFC was conducted in *Nicotiana benthamiana* for testing interaction between MdSUT2.2 and MdCIPK22. the tonoplast marker (AtCBL3‐RFP) was carried. (Scale bars: 100 μm); (b) *In vitro* GST pull‐down assays between MdSUT2.2 and MdCIPK22. MdCIPK22‐His proteins were incubated with immobilized MdSUT2.2‐GST or GST, and the proteins immunoprecipitated with His‐beads were detected using anti‐GST antibody; (c) *In vivo* Co‐IP assays between MdSUT2.2 and MdCIPK22. The total proteins were extracted from 35S::MdSUT2.2‐Myc+35S::MdCIPK22‐HA or 35S::MdSUT2.2‐Myc+35S::HA co‐expressed apple calli, and then immunoprecipitated with anti‐HA antibody. The proteins from crude lysates (input) were detected with anti‐Myc antibody. The immunoprecipitated proteins (output) were detected with anti‐HA antibody.

To elucidate the molecular mechanism by which drought induces phosphorylation modification for MdSUT2.2 protein, a DUALmembrane system for yeast two‐hybridization screening was performed to screen through an apple cDNA library for MdSUT2.2‐interacting protein. As a result, several positive colonies were obtained. Sequence analysis revealed that one of these colonies contained a cDNA fragment which encodes a part of a CIPK protein. Protein sequence alignment demonstrated that this protein is closely related with AtCIPK22, thereby named as MdCIPK22 hereafter (Figure S9). Structure domain analysis showed that MdCIPK22 has an activation loop and a NAF domain (Figure S10). Their interaction was further confirmed by BiFC assays in leaf of *Nicotiana benthamiana* with an agroinfiltration method. The result showed that green fluorescence indicative of heterodimer formation of MdSUT2.2‐YFPn and MdCIPK22‐YFPc was observed (Figure 4a). Green fluorescence was co‐localized with a tonoplast protein AtCBL3‐RFP (Figure 4a), indicating that MdCIPK22 interacted with MdSUT2.2 on the tonoplast.

Subsequently, pull‐down assay was conducted to confirm the interaction between MdCIPK22 and MdSUT2.2 *in vitro*. MdCIPK22‐His and MdSUT2.2‐GST recombinant proteins were purified and used for GST pull‐down assays. The result showed that MdCIPK22‐His was pulled down by MdSUT2.2‐GST fusion protein, but not by GST (Figure 4b), indicating that MdCIPK22 physically interacted with MdSUT2.2 *in vitro*. In addition, it was found that S‐to‐A mutation in GST‐labeled MdSUT2.2^S381A^ protein did not influence the interaction between MdCIPK22 and MdSUT2.2 (Figure S11).

To further verify their interaction *in vivo*, Co‐IP assays were performed using 35S::MdSUT2.2‐Myc/35S::HA and 35S::MdSUT2.2‐Myc/35S::MdCIPK22‐HA transgenic calli. Total proteins were isolated from two kinds of transgenic calli and immunoprecipitated (IPed) with an anti‐Myc antibody. The IP‐ed protein samples were used for immunoblotting assay with an anti‐HA antibody. The result showed that MdCIPK22‐HA was Co‐IPed by anti‐Myc antibody in 35S::MdSUT2.2‐Myc/35S::MdCIPK22‐HA transgenic calli, but HA was not in 35S::MdSUT2.2‐Myc/35S::HA calli (Figure 4c), indicating that MdCIPK22 interacted with MdSUT2.2 in *in vivo* cells.

### MdCIPK22 proteins are required for phosphorylation of MdSUT2.2 proteins at Ser^381^

**Figure 5:**
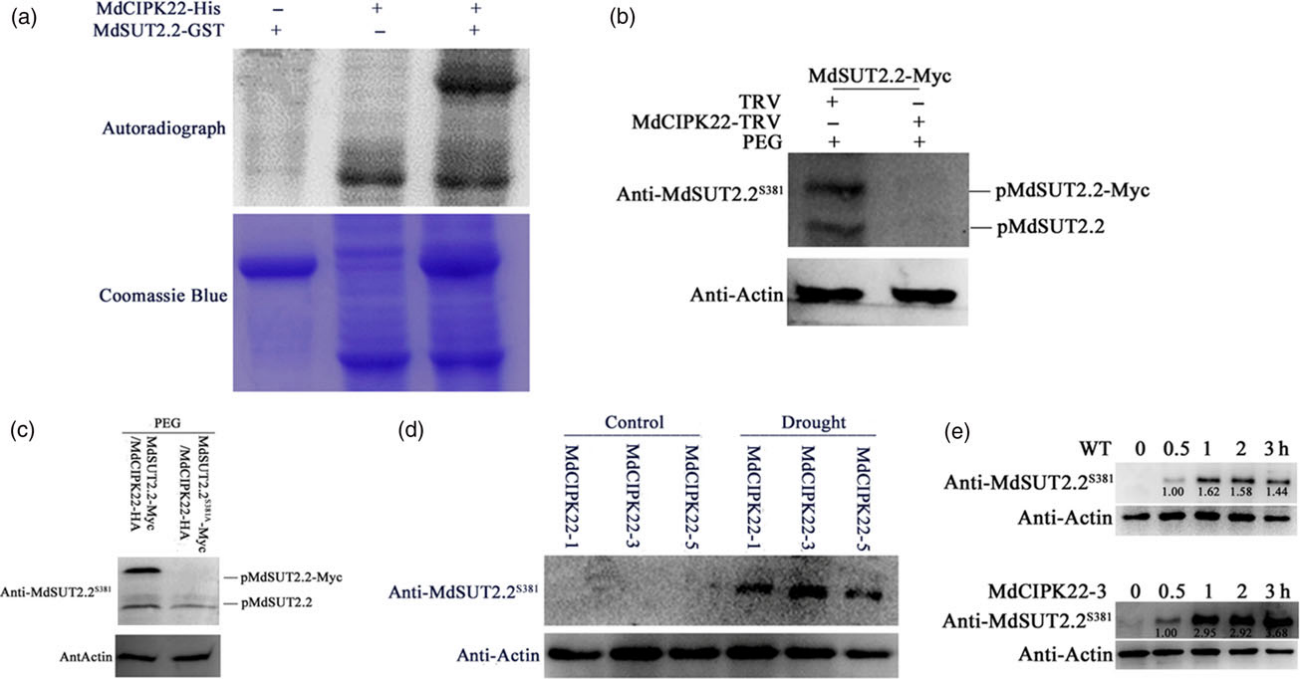
MdCIPK22 is required for drought‐induced phosphorylation of MdSUT2.2 protein. (a) *In vitro* phosphorylation assay of MdSUT2.2 by protein kinase MdCIPK22. SDS‐PAGE gel with coomassie blue‐stained MdCIPK22‐His, MdSUT2.2‐GST proteins (bottom panel); autoradiograph showing MdSUT2.2‐GST phosphorylation by MdCIPK22‐His (top panel, top band) and MdCIPK22‐His autophosphorylation (top panel, bottom bands). (b) MdSUT2.2 phosphorylation was examined in 35S::MdSUT2.2‐Myc transgenic apple calli which was infected with or without a viral based vector MdCIPK22‐TRV after PEG treatment for 1 h. TRV empty vector‐infected apple calli were used as the control. Western blotting assays were performed with anti‐MdSUT2.2^S381^ antibody; (c) MdSUT2.2 phosphorylation in PEG‐treated MdCIPK22‐HA/MdSUT2.2‐Myc and MdCIPK22‐HA/MdSUT2.2^S381A^‐Myc calli with an anti‐MdSUT2.2^S381^ antibody; (d) MdSUT2.2 phosphorylation in three overexpression lines MdCIPK22‐1, MdCIPK22‐3, and MdCIPK22‐5 treated with or without drought using anti‐MdSUT2.2^S381^ antibody; (e) Western blot assays using anti‐MdSUT2.2^S381^ antibody on MdSUT2.2 phosphorylation in transgenic line MdCIPK22‐3 and the WT control, which were treated with drought at different time.

In vitro phosphorylation assays were performed using recombinant proteins MdSUT2.2‐GST and MdCIPK22‐His to examine if MdSUT2.2 is a direct substrate of MdCIPK22. The result showed that MdSUT2.2‐GST fusion protein was phosphorylated by MdCIPK22‐His (Figure 5a), indicating that MdSUT2.2 protein is a direct substrate of MdCIPK22 protein kinase. To determine whether phosphorylation of MdSUT2.2 protein needs MdCIPK22 protein, a viral vector MdCIPK22‐TRV was obtained and used to infect 35S::MdSUT2.2‐Myc transgenic calli, while empty vector tobacco rattle virus (TRV) was used as the control (Figure S12). The resultant double transgenic calli 35S::MdSUT2.2‐Myc/MdCIPK22‐TRV and 35S::MdSUT2.2‐Myc/TRV were used for Western blotting assay with phospho‐specific anti‐MdSUT2.2^S381^ antibody. The result showed that both endogenous MdSUT2.2 and exogenous MdSUT2.2‐Myc were phosphorylated in 35S::MdSUT2.2‐Myc/TRV calli treated with PEG. In contrast, *MdCIPK22* silencing disrupted PEG‐induced phosphorylation of MdSUT2.2 and MdSUT2.2‐Myc in 35S::MdSUT2.2‐Myc/MdCIPK22‐TRV calli (Figure 5b), indicating that PEG (or drought)‐induced phosphorylation of MdSUT2.2 protein depended on MdCIPK22 protein.

To further examine if residue Ser^381^ is crucial for MdCIPK22‐meidated phosphorylation of MdSUT2.2 protein, expression vector 35S::MdCIPK22‐HA was genetically introduced into 35S::MdSUT2.2‐Myc and 35S::MdSUT2.2^S381A^ calli respectively. As a result, double transgenic calli MdSUT2.2‐Myc/MdCIPK22‐HA and MdSUT2.2^S381A^‐Myc/MdCIPK22‐HA were obtained (Figure S13) and used to examine phosphorylation with phospho‐specific anti‐MdSUT2.2^S381^ antibody. The result showed that MdCIPK22 phosphorylated both MdSUT2.2 and MdSUT2.2‐Myc in MdSUT2.2‐Myc/MdCIPK22‐HA calli (Figure 5c). However, MdCIPK22 phoshorylated MdSUT2.2 but not MdSUT2.2^S381A^‐Myc in MdSUT2.2^S381A^‐Myc/MdCIPK22‐HA calli (Figure 5c). These results indicated that Ser^381^ is a crucial site for MdCIPK22‐mediated phosphorylation of MdSUT2.2 protein in reponse to drought stress.

To examine if MdCIPK22‐mediated phosphorylation of MdSUT2.2 protein is drought‐dependent, expression vector 35S::MdCIPK22‐Myc were constructed and used to transform apple. As a result, 3 independent transgenic lines MdCIPK22‐1, MdCIPK22‐3 and MdCIPK22‐5 were obtained and treated with or without drought (Figure S14a,b). Protein samples were extracted from three transgenic lines, respectively, and used for phosphorylation assay with phospho‐specific anti‐MdSUT2.2^S381^ antibody. The result showed that drought treatment is required for MdCIPK22‐mediated phosphorylation of MdSUT2.2 protein (Figure 5d). In addition, phosphorylation level of MdSUT2.2 protein increased with duration of drought treatment (Figure 5e), just like in calli (Figure 3f). In addition, it was found that the phosphorylation intensity of MdSUT2.2 proteins increased faster in transgenic MdCIPK22‐3 than in WT with the treatment duration (Figure 5e). These results showed that MdCIPK22 increased phosphorylation degree of MdSUT2.2 under drought treatment.

### MdCIPK22‐mediated phosphorylation stabilizes MdSUT2.2 proteins

**Figure 6:**
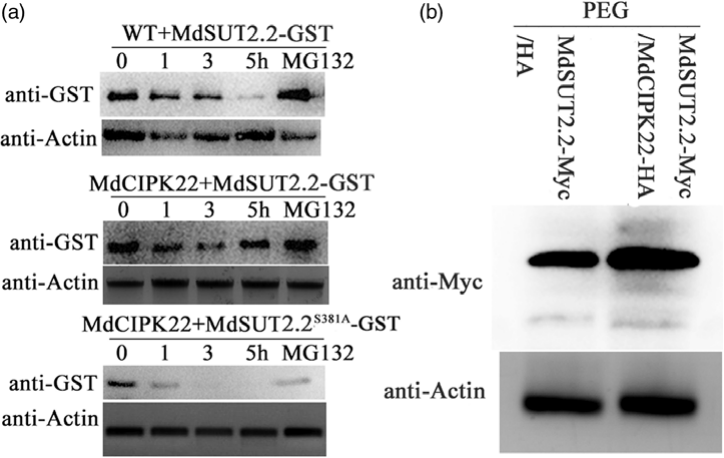
MdCIPK22‐mediated phosphorylation stabilizes MdSUT2.2 protein. (a) Cell‐free degradation assay of recombinant MdSUT2.2‐GST or MdSUT2.2^S381A^‐GST proteins in the protein extracts of transgenic apple calli, as labeled. Actin was used as a loading control to ensure identity in the amounts of protein. Protein levels of MdSUT2.2‐GST and MdSUT2.2^S381A^‐GST were visualized by immunoblotting using the anti‐GST antibody; (b) MdSUT2.2 abundancy *in vivo* in MdSUT2.2‐Myc/HA and MdSUT2.2‐Myc/MdCIPK22‐HA transgenic calli treated with drought.

Cell‐free degradation assays were carried out to test how MdCIPK22 affect MdSUT2.2. MdSUT2.2‐GST and MdSUT2.2^S381A^‐GST fusion proteins were expressed and purifed in *Escherichia coli*, followed by an addition of total proteins extracted from the WT and 35S::MdCIPK22‐HA calli respectively. Protein gel blotting with anti‐GST antibody showed that MdSUT2.2‐GST proteins degraded slower in total proteins extracted from 35S::MdCIPK22‐HA transgenic calli than in those from the WT control (Figure 6a). Meanwhile, MdSUT2.2^S381A^‐GST degraded much faster than MdSUT2.2‐GST proteins (Figure 6a). In addition, MG132 noticeably inhibited degradation of MdSUT2.2‐GST and MdSUT2.2^S381A^‐GST proteins (Figure 6a). These results suggested that MdSUT2.2 degraded probably via 26S proteasome and that MdSUT2.2 phosphorylation at Ser^381^ inhibited this process.

Subsequently, MdSUT2.2 protein abundancy was examined with anti‐Myc antibody in MdSUT2.2‐Myc/HA and MdSUT2.2‐Myc/MdCIPK22‐HA transgenic calli treated with PEG. It was found that MdSUT2.2 abundancy was greater in MdSUT2.2‐Myc/MdCIPK22‐HA calli than in MdSUT2.2‐Myc calli (Figure 6b), indicating that MdCIPK22 stabilized MdSUT2.2 proteins *in vivo*.

### MdCIPK22 improves drought tolerance in an MdSUT2.2‐dependent manner

**Figure 7:**
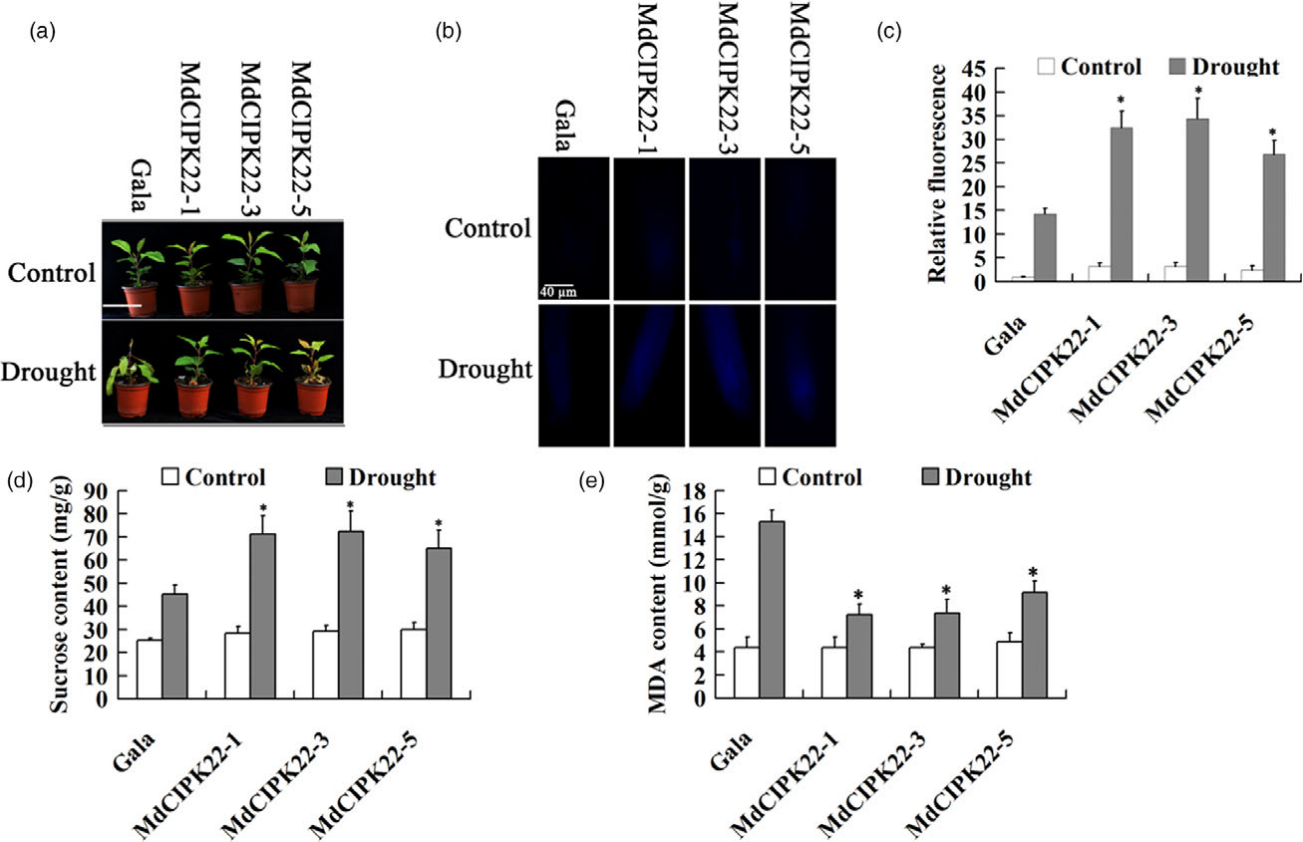
MdCIPK22 overexpression increases sucrose content and enhances drought tolerance in transgenic apple plants. (a) MdCIPK22 transgenic plants exhibits enhanced drought tolerance; (b) Esculin uptake assay of sucrose transport activity in root of transgenic plants MdCIPK22‐1, MdCIPK22‐3 and MdCIPK22‐5; (c) The relative fluorescence intensity of (b); (d) and (e) Sucrose content in dry weight and MDA content in fresh weight of transgenic and the WT control plants treated with or without drought. n.s., *P* > 0.01;*P < 0.01;**P < 0.001.

Three independent transgenic lines MdCIPK22‐1, MdCIPK22‐3 and MdCIPK22‐5 were treated with drought stress for tolerance analysis (Figure 7a). It was found that they exhibited higher sucrose transport activity into vacuole as indicated by esculin uptake both in root than the WT control under drought stress (Figure 7b,c). Moreover, they accumulated much more sugars under drought stress (Figure 7d). Finally, it was found that three transgenic lines were more tolerant to drought stress, as manifested by less leaf‐wilting symptoms and less MDA content than the WT control (Figure 7a,e). These data indicated that MdCIPK22 functions as a positive regulator in response to drought stress.

**Figure 8:**
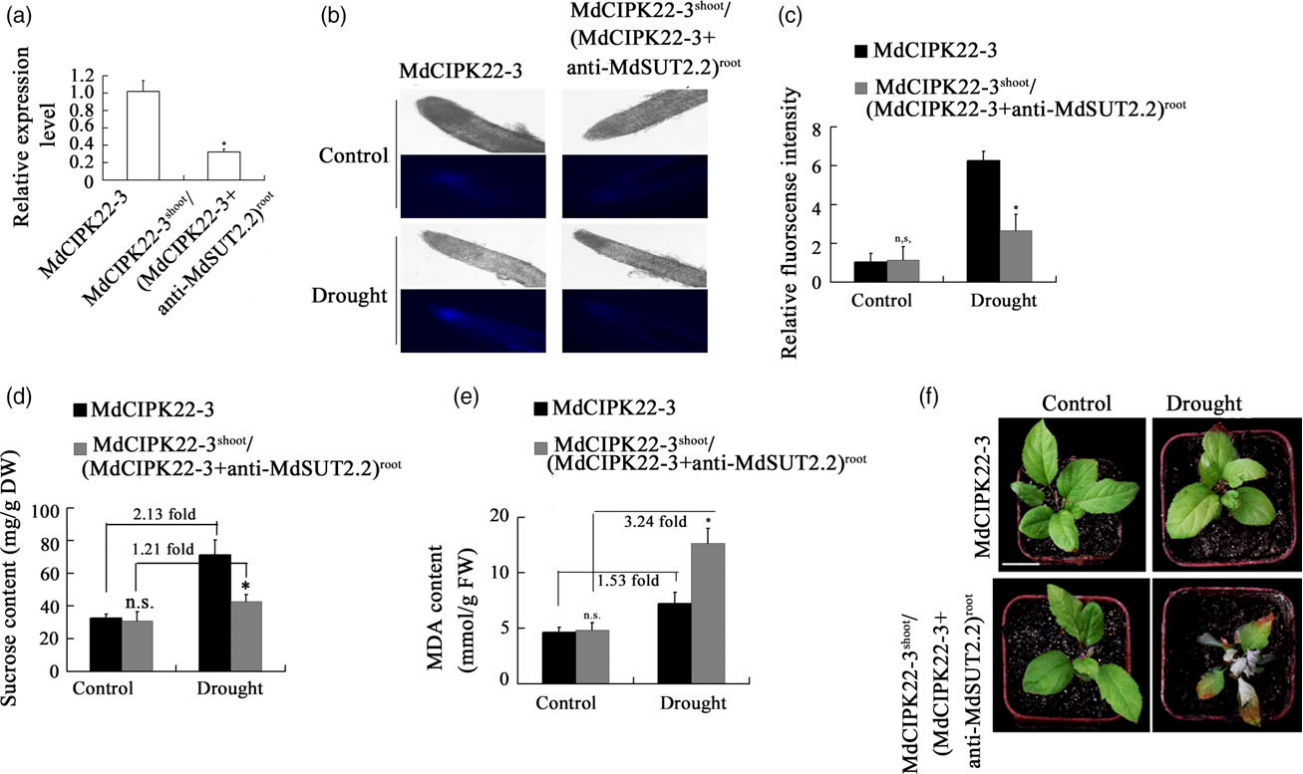
MdCIPK22 increases sucrose accumulation and drought tolerance in a MdSUT2.2‐dependent manner. (a) Expression levels of *MdSUT2.2* in MdCIPK22‐3 and MdCIPK22‐3^shoot^/(MdCIPK22‐3 + anti‐MdSUT2.2)^root^ transgenic plants; (b) Esculin uptake assay of sucrose transport activity in roots of MdCIPK22‐3 and MdCIPK22‐3^shoot^/(MdCIPK22‐3 + anti‐MdSUT2.2)^root^ transgenic plant; (c) The relative fluorescence intensity of (B); (d‐e) Sucrose content in dry weight and MDA　content in fresh weight in roots of MdCIPK22‐3 and MdCIPK22‐3^shoot^/(MdCIPK22‐3 + anti‐MdSUT2.2)^root^ transgenic plants; (f) Drought tolerance observation of MdCIPK22‐3 and MdCIPK22‐3^shoot^/(MdCIPK22‐3 + anti‐MdSUT2.2)^root^ transgenic plants; Values are the mean of three replicates, and differences with a *P*‐value < 0.05 were considered significant. n.s., *P* > 0.01;*P < 0.01;**P < 0.001.

To examine if MdSUT2.2 is required for MdCIPK22‐mediated improvement in drought tolerance, the expression of *MdSUT2.2* gene was specifically suppressed in the root of MdCIPK22‐3 transgenic plant using an *Agrobacterium rhizogenes*‐mediated method. The successfully transformed roots were distinguished with red fluorescence (Figure S15). Expression analysis demonstrated that the expression of *MdSUT2.2* gene was specifically and noticeably down‐regulated in transformnant roots (Figures 8a and S16), indicating that a chimeric transgenic plant with *MdCIPK22* overexpression in shoot and *MdCIPK22* overexpression plus *MdSUT2.2* suppression in root was obtained and referred as MdCIPK22‐3^shoot^/(MdCIPK22‐3 + anti‐MdSUT2.2)^root^. Then, plantlets of transgenic line MdCIPK22‐3 and MdCIPK22‐3^shoot^/(MdCIPK22‐3 + anti‐MdSUT2.2)^root^ were treated with drought for 20 days. Esculin uptake assays demonstrated that MdCIPK22‐3^shoot^/(MdCIPK22‐3 + anti‐MdSUT2.2)^root^ plantlets exhibited a lower sucrose transport activity both in root than MdCIPK22‐3 under drought stress (Figure 8b,c). As a result, plantlets accumulated less sucrose in root than MdCIPK22‐3 under drought stress (Figure 8d). The drought‐treated line MdCIPK22‐3 accumulated 2.13‐fold sucrose, while the drought‐treated MdCIPK22‐3^shoot^/(MdCIPK22‐3 + anti‐MdSUT2.2)^root^ plants just did 1.21 fold sucrose, comparing to the corresponding non‐treatment controls respectively (Figure 8d). Finally, it was found that MdCIPK22‐3^shoot^/(MdCIPK22‐3 + anti‐MdSUT2.2)^root^ plantlets generated more MDA in root and contained lower relative water content than MdCIPK22‐3 plantlets (Figure 8e,f,g), indicating that MdCIPK22‐mediated sucrose accumulation and drought tolerance required sucrose transporter MdSUT2.2 at least partially, if not all.

## Discussion

Drought stress causes premature plant death, affects the plant growth and development. In this study, it was found that a protein kinase MdCIPK22 positively regulated drought tolerance by interacting with and phosphorylating MdSUT2.2 proteins. These findings yield new insight into the molecular mechanism by which drought and the related stresses induce sugar accumulation in plant.

Sugar accumulation has long been reported to be a part of the drought‐stress responses, and occurs at very high levels when plants experience conditions of drought stress (Szabados and Savoure, 2010). Environmental stimuli such as drought stress promote sugar accumulation by inducing the expression of sugar biosynthesis and transport genes, which is very important for enhancing stress tolerance (Ferrandino and Lovisolo, 2014). Sucrose transporters (SUTs/SUCs) are proton‐coupling sucrose uptake transporters, and have been isolated and functionally characterized in various plant species (Barth *et al*., 2003; Kühn *et al*., 2003; Schneider *et al*., 2012; Shitan and Yazaki, 2013; Srivastava *et al*., 2008). The expression of *SUTs* (or *SUCs*) and *TMTs* genes are induced by various stresses (Wormit *et al*., 2006). Transcripts of *Arabidopsis AtSUCs* and rice *OsSUT2* genes are up‐regulated in response to low temperatures, drought and salinity stresses (Ibraheem *et al*., 2011; Lundmark *et al*., 2006). *AtSUC2* and *AtSUC4* are induced by salt, osmotic, low temperature and exogenous ABA treatments and are required for abiotic stress tolerance in an ABA‐dependent manner (Gong *et al*., 2015). Furthermore, a series of transgenic analysis demonstrated that transgenic plants ectopically or overly expressing *SUTs* or *SUCs* genes resulted in increased sucrose and soluble sugar accumulation, as well as exhibited promoted stress tolerance (Kühn *et al*., 2003; Ma *et al*., 2016). Here, it was found that the expression level of apple sugar transport genes *MdSUT2.1* and *MdSUT2.2* increased with drought stress. Overexpression of *MdSUT2.2* increased sugar contents and enhanced drought tolerance (Figure 2). In apple, *MdSUT2.2* has sucrose transporter activity and functions as a sucrose transporter (Ma *et al*., 2017a).

Sugars act not only as energy or osmotic adjustment compound, but also as signalling molecules that are involved in the regulation of plant development and growth (Srivastava *et al*., 2008). For example, a high concentration of sugars triggers the repression of genes associated with photosynthesis (Hammond *et al*., 2011). Sugar also regulates anthocyanin biosynthesis. In *Arabidopsis*, sucrose induces the expression of *MYB75/PAP1* gene, which activates the expression of structural genes associated with anthocyanin synthesis (Solfanelli *et al*., 2006). The most recently, it is found that apple glucose sensor MdHXK1 interacts with and phosphorylates a bHLH transcrpition factor to regulate anthocyanin accumulation (Hu *et al*., 2016).

In fact, SUT2 shows many features similar to the yeast sugar sensors SNF3 and RGT2 and is considered as sucrose sensor (Barker *et al*., 2000). It influences sucrose fluxes across the plasma membrane of sieve elements by directly controlling SUT1 and SUT4 (Barker *et al*., 2000). Mutation to sucrose transporter *AtSUC1* results in a reduced ethylene production in response to sucrose and light in *Arabidopsis* (Jeong *et al*., 2010). Arabidopsis SUC2 and SUC4 may be involved in sucrose signalling under abiotic stress conditions. A few of stress‐responsive genes such as *RD29A*,* RD29B*,* ABI1* and *CHS* are inhibited in mutant *suc2* and *suc4* (Gong *et al*., 2015). Ectopic expression of an apple *MdSUT2.2* gene leads to early flowering in transgenic *Arabidopsis* (Ma *et al*., 2016). Therefore, MdSUT2.2 involves in responses to abiotic stresses not only by transporting sucrose, but also by sensing sugar signal. No matter sucrose sensor and transporter, SUT2 is crucial component in drought or other stress signalling.

In addition, apple protein kinase MdCIPK22 was isolated and characterized with function in the positive regulation of drought tolerance. CIPK proteins form a calcium decoding signalling network and play an important role in plant responses to abiotic stresses. In *Arabidopsis*, AtCIPK6 mediates an ER‐targeted calcium‐binding peptide to confer salt and drought tolerance (Tsou *et al*., 2012). In maize, ZmCIPK8 is involved in plant responses to drought and other abiotic stresses by regulating stress‐related genes (Tai *et al*., 2016). The expression of the *P5CS1* gene is regulated by OsCIPK1, enhancing production of proline which acts as a cryoprotectant during cold stress and as osmolyte during drought stress in rice (Abdula *et al*., 2016). Both wheat TaCIPK2 and *Stipa purpurea* SpCIPK26 positively regulate the expression CAT genes, thereby negatively modulating H_2_O_2_ content (Wang *et al*., 2016; Zhou *et al*., 2016). TaCIPK27 interacts with calcineurin B‐like (CBL) proteins and positively regulates drought tolerance (Wang *et al*., 2016). In this study, it was found that *MdCIPK22* transgenic apple plant produced more sugars and exhibited higher drought tolerance than the WT control (Figures 7 and 8).

Protein kinase plays an important role in protein posttranslational modification and is involved in the regulation of transporter activity (Krügel *et al*., 2008; Schulze *et al*., 2000). In this study, it was found that apple protein kinase MdCIPK22 interacted with and phosphorylated MdSUT2.2 to increase its stability in response to drought (Figures 4 and 6). MdSUT2.2 protein is phosphorylated by MdCIPK22 at a serine at site 381 (Figure S17). Generally, the extended cytoplasmic loops (CCB1 and CCB2 domains) of SUT2 proteins contain a serine residue at a highly conserved position, which is predicted to be a target for phosphorylation (Figure S17; Roblin *et al*., 1998; Barker *et al*., 2000). However, our study showed that the serine is not in the conserved CCB1 and CCB2 domains. The 381 serine residue was a novel phosphorylation site (Figure S17). A mutation to this site led to a completely loss of phosphorylation for MdSUT2.2 protein (Figure 3d), indicating it is a single novel and specific site for phoshporylation in SUT2 proteins in response to drought stress.

For fruit crops, Apple is a world‐widely planted fruit tree. Sugar accumulation in the vacuole determines fruit sweetness and improves fruit quality (Ma *et al*., 2016; Shiratake and Martinoia, 2007). Sweetness plays an important role in fruit quality of apple and other fruit trees. Generally, moderate drought stress improves fruit quality by enhancing sugar accumulation in fruits (Barry *et al*., 2004). Therefore, MdCIPK22‐MdSUT2.2 regulatory module shed light to the molecular mechanism by which plants respond to drought stress and drought improves fruit quality. It provides a new idea for further attempt to improve stress tolerance and fruit quality by cultivation and breeding methods.

## Materials and methods

### Plant materials, growth conditions and drought treatments

Tissue cultures of *Malus × domestic cv*. ‘Royal Gala’ apples were used as the wild type. The *in vitro* shoot cultures of apple cultivar ‘Gala’ were grown on MS medium supplemented with 1.0 mg/L naphthyl acetate (NAA) and 0.5 mg/L 6‐benzylaminopurine (6‐BA) at 25 °C under long‐day conditions (16 h light/8 h dark). They were subcultured at 30‐day intervals. For drought treatment, apple shoot cultures were cultivated on MS medium supplemented with 0.5 mg/L indole‐3‐acetic acid (IAA), 1.5 mg/L 6‐benzylaminopurine (6‐BA) and 6% polyethylene glycol (PEG6000, imitating drought).

The rooted apple plantlets were transferred to pots containing a mixture of soil/perlite (1 : 1) and grown in the greenhouse under a 16 h/8 h light/dark and 25 °C day/night cycle. For drought treatment, plantlets with the same growth state were imposed by withholding water for 20 days. For phosphorylation detection, plants was treated under drought conditions for 20, 40, 60 min.

The apple calli used in this study were induced from the young embryos of ‘Orin’ apples (*Malus domestica* Borkh.). They were grown on MS medium supplemented with 0.5 mg/L indole‐3‐acetic acid (IAA) and 1.5 mg/L 6‐benzylaminopurine (6‐BA) at 25 °C in the dark. The calli were subcultured three times at 15‐day intervals before being used for genetic transformation and in other assays. For drought treatment, wild‐type apple calli and transgenic apple calli were grown on MS medium supplemented with 0.5 mg/L indole‐3‐acetic acid (IAA), 1.5 mg/L 6‐benzylaminopurine (6‐BA) and 6% PEG6000 at 25 °C in the dark for 20 days.

### Construction of expression vectors and genetic transformation

To construct sense overexpression vectors, sense full‐length cDNAs of MdSUT2.2 and MdCIPK22 were amplified respectively. The resultant PCR products were inserted into the pBI121 vector under the control of the 35S promoter. As a result, residue Ser381 was replaced with Ala381 in MdSUT2.2 protein. The mutated MdSUT2.2^S381A^ was then inserted downstream CaM 35S promoter into overexpression vector pBI121. These vectors were genetically introduced into the ‘Orin’ calli and apple culture seedlings using Agrobacterium‐mediated transformation as described previously (Xie *et al*., 2012). All of the primers used in this study are listed in Table S1.

### RNA extraction and qRT‐PCR assays

Total fruit RNA was extracted with the hot borate method as described by Xie *et al*. (2012). Total RNA from other tissues was extracted with the Trizol Reagent (Invitrogen Life Technologies, Carlsbad, CA). Two micrograms of total RNA were used to synthesize first strand cDNA with a PrimeScript First Strand cDNA Synthesis Kit (TaKaRa, Dalian, China). The primer used to generate cDNA is oligo DT.

For real‐time quantitative PCR (qRT‐PCR) analysis, the reactions were performed with iQ SYBR Green Supermix in an iCycler iQ5 system (Bio‐Rad, Hercules, CA) following the instructions of the manufacturer. The relative quantification of specific mRNA levels was performed using the cycle threshold (*C*_t_) 2^−DDCt^ method (Software IQ5 2.0). For all analyses, the signal obtained for a gene of interest was normalized against the signal obtained for the 18S gene. All of the samples were tested in three biological replicates. *t*‐test was used to analyse the numerical data with Microsoft Excel (2003) and SPSS statistical software 13.0 (SPSS Inc., Chicago, US).

### Protein extraction and Western blotting

Approximately, 500 mg of apple calli and shoot cultures were ground in a buffer containing 100 mm Tris (pH 8.0), 1 mm EDTA, 0.1% (w/v) PVP, 10 mm b‐ME, 200 mm sucrose and 0.5% (w/v) protease inhibitor mixture (Sigma Aldrich, St. Louis, MO). After homogenization, the mixture was clarified though centrifugation, and the protein concentration was determined using the Bradford Reagent (Sigma‐Aldrich) with BSA as a standard.

Anti‐Myc monoclonal antibodies and phosphorylated antibodies anti‐MdSUT2.2^S381^ were prepared by GenScript Company (Nanjing, China). MdSUT2.2 and MdCIPK22 protein levels were determined by protein gel blotting using an anti‐MYC antibody. Protein extracts were separated on a 12% SDS‐PAGE gel and transferred to PVDF membranes (Roche, Indianapolis, IN) using an electrotransfer apparatus (Bio‐Rad). The membranes were incubated with anti‐MYC, or anti‐pMdSUT2.2^S381^ primary antibodies and then peroxidase‐conjugated secondary antibodies (Abcam, Shanghai, China) before visualization of immunoreactive proteins using an ECL detection kit (Millipore, Billerica, MA). ACTIN served as a protein loading control. Each value represents the mean ± SD from three separate experiments.

### Co‐immunoprecipitation procedures

For the IP assays, 1 mg of freshly extracted proteins from 35S::MdSUT2.2‐Myc+35S::MdCIPK22‐HA or 35S::MdSUT2.2‐Myc+35S::HA co‐expressed apple calli were precleaned with 30 mL of Protein A agarose beads (4 h, 4 °C). The beads were centrifuged, and the supernatant was transferred into a fresh tube and incubated with anti‐MYC antibody (overnight, 4 °C). After brief centrifugation, four washing steps followed, after which loading buffer was added to the precipitates and boiled. For CO‐IP analysis, precipitates were further analysed by SDS‐PAGE and Western Blotting as described by Ma *et al*. (2017b). The proteins from crude lysates (input) were detected with anti‐Myc antibody, and the IPed proteins (output) were detected with anti‐HA antibody. MdCIPK22‐HA protein can be detected if it interacted with MdSUT2.2‐Myc with anti‐HA antibody. HA protein was used as negative control.

### Pull‐down analysis

The full‐length cDNAs of MdCIPK22 was amplified by PCR with additional restriction enzyme sites and inserted into the *Eco*RI‐*Sal*I sites of pET‐32a to produce a His‐tagged recombinant protein. The full‐length MdSUT2.2 coding regions were cloned into the *Eco*RI‐*Sal*I sites of pGEX‐4T‐1 to produce a GST fusion protein. For protein expression, the plasmids were transformed into *E. coli* BL21 (DE3) (Transgene, Beijing, China) and induced with 0.1 mm isopropyl β‐D‐1‐thiogalactopyranoside (IPTG) in Luria‐Bertani broth for 6 h at 16 °C. For pull‐down analysis with the GST‐ and His‐tagged proteins, MdSUT2.22‐GST proteins were eluted from glutathione‐agarose beads before incubation with MdCIPK22‐His that remained attached to the tetradentate‐chelated nickel resin. In general, proteins were incubated for at least 4 h at 4 °C with shaking before being centrifuged. Precipitates were washed no less than three times to remove nonspecific binding and boiled (10 min, 100 °C).

### Malondialdehyde level

The leaves were cut into several segments in 5 mL of 10% phosphate buffered saline (PBS) and centrifuged at 12 000 *g* for 10 min. Two millilitres of the supernatant was added to 5 mL of 0.5% thiobarbituric acid (TBA, in 10% TCA), and the reaction mixture was incubated at 100 °C in a water bath for 10 min. The reaction was cooled to room temperature, and the absorbance of the supernatant at 450, 532 and 600 nm was determined using an UVevis spectrophotometer (UV‐2450). Thiobarbituric acid was used as a blank. The following formula was used to estimate MDA levels: MDA content (mmol/g FW) ¼ [6.542*(OD532‐OD600)‐0.559*OD450] (mmol L1)*V (mL)/fresh weight (g FW). *t*‐test was used to analyse the numerical data with Microsoft Excel (2003) and SPSS statistical software 13.0 (SPSS Inc.). Each value represents the mean ± SD from three separate experiments.

### Esculin uptake

Plants root were then rinsed and mounted on glass slides in ½ strength MS liquid media with esculin under normal or drought conditions. The fluorescence was measured using a ZEISS LSM880 spectrofluorometer with a 367 nm excitation wavelength and a 454 nm emission wavelength. *t*‐test was used to analyse the numerical data with Microsoft Excel (2003) and SPSS statistical software 13.0 (SPSS Inc.).

### Cell‐free degradation

Cells (*E. coli,* BL21) were induced by 0.1 mm IPTG and allowed to grow for 4 h at 16 °C. MdSUT2.2‐GST protein was eluted from the tetradentate‐chelated nickel resin. The total proteins of the transgenic apple calli were subsequently extracted in degradation buffer containing 25 mm Tris‐HCl, pH 7.5, 10 mm NaCl, 10 mm MgCl_2_, 4 mm PMSF, 5 mm DTT, and 10 mm ATP. The supernatant was collected, and the protein concentration was determined by the using the Bradford assay reagent (Bio‐Rad). Each reaction mix contained 100 ng of MdSUT2.2‐GST and 500 μg of total protein from transgenic apple calli. For the proteasome inhibitor experiments, 20 μm MG132 was added 30 min prior to the experiment. The reaction mixes were incubated at 22 °C, and the reactions were stopped by the addition of SDS‐PAGE sample buffer and boiling (10 min, 100 °C). The results were quantified using Quantity One 1‐D Analysis Software (Bio‐Rad). Each value represents the mean ± SD from three separate experiments.

### The DUALmembrane system

The DUAL membrane system takes advantage of the split‐ubiquitin mechanism to measure the interaction between an integral membrane protein and its interaction partners (Thaminy *et al*., 2003). MdSUT2.2 is fused to the bait vector pBT3‐STE. Then the resulting vector was transformed into the reporter strain NMY51 according to the transformation procedure. The successful transformation was plated onto selective SD/‐Leu medium. Western blotting was performed to verify expression by antibody directed against LexA. The cDNA library is fused to NubG to generate the prey. Seven microgram library plasmid was transformed into yeast strain NMY51 expressing the bait MdSUT2.2. using a lithium acetate method. Yeast cells were cultured on minimal medium ‐Leu/‐Trp according to the manufacturer's instructions. Transformed colonies were plated onto minimal medium ‐Leu/‐Trp/‐His/‐Ade with or without β‐galactosidase to test for possible interactions.

### Construction of the viral vectors and transient expression in apple calli

To construct antisense expression viral vectors, an MdSUT2.2 fragment were amplified with PCR using apple cDNA as the template. The resultant products were cloned into the TRV vector, in the antisense orientation under the control of the dual 35S promoter. The vector was named MdSUT2.2‐TRV. All of the vectors were transformed into *Agrobacterium tumefaciens* strain GV4404 for inoculations. The calli and apple leaves infection were performed as previously described (Hu *et al*., 2016).

### *Agrobacterium rhizogenes*‐mediated transformation of *MdUT2* into root

A specific fragment of MdSUT2.2 was amplified with primers MdSUT2.2‐F: CACCGAGCTCTTTGGGTGTCGGATGC and MdSUT2.2‐R: CTGACTCAGTAATGGCCGCGACTCA. The resultant cDNA fragment was cloned into pK7GWIWG2 vector. pK7GWIWG2 vector contained a ubiquitin promoter‐driven red fluorescent protein gene (DsRed1) and spectinomycin resistance gene. The resultant vector pK7GWIWG2‐MdSUT2.2‐RFP was transformed into *A. rhizogenes* strain MSU440 and used for infection. Shoot cultures of transgenic apple line MdCIPK22‐3 with the same growth state were used for infection as described by Xiao *et al*. (2014). Then, the infected shoot cultures were grown in MS medium plus 0.2 mg/L IAA to induce roots.

The rooted apple plantlets were transferred to pots containing a mixture of soil/perlite (1 : 1) and grown in the greenhouse under a 16 h/8 h light/dark and 25 °C day/night cycle for 30 days.

## Author contributions

Yu‐Jin Hao and Qi‐Jun Ma conceived and designed the experiment; Qi‐Jun Ma, Mei‐Hong Sun, Jing Lu, Hui Kang and Chun‐Xiang You preformed the experiments; Qi‐Jun Ma and Yu‐Jin Hao analysed the data and wrote the paper.

## Conflict of interest

The authors declare no conflict of interest.

## Supporting information


**Figure S1** The phylogenetic tree analysis of MdSUTs and AtSUTs.
**Figure S2** The sequence similarity of AtSUT2, MdSUT2.1 and MdSUT2.2.
**Figure S3** The expression of MdSUT2.1 and MdSUT2.2 was induced by drought.
**Figure S4** The transmembrane domains of AtSUT2.
**Figure S5** Soluble sugar content and relative water content in three transgenic lines (MdSUT2.2‐1, MdSUT2.2‐2 and MdSUT2.2‐5) and the WT ‘Gala’ control treated with or without drought in plant dry weight.
**Figure S6** Collision‐induced dissociation mass spectrum showed the phosphorylation site was serine (S) at residue 381 (S381) of the MdSUT2.2 protein.
**Figure S7** PEG induces phosphorylation for MdSUT2.2 protein in the wild type apple calli.
**Figure S8** PEG tolerance of transgenic calli (MdSUT2.2‐Myc and MdSUT2.2^S381A^‐Myc) and the WT control.
**Figure S9** Amino acid sequence alignment of MDP0000154855 and AtCIPK22.
**Figure S10** Functional domain analysis of CIPK proteins in different plant species.
**Figure S11** Pull‐Down assay of protein interaction between MdSUT2.2^S381A^‐GST and MdCIPK22‐His.
**Figure S12** Expression level of *MdCIPK22* gene in 35S::MdSUT2.2‐Myc/MdCIPK22‐TRV and 35S::MdSUT2.2‐Myc/TRV calli.
**Figure S13** Expression level of *MdCIPK22* and MdSUT2.2 gene were detected in MdSUT2.2‐Myc/MdCIPK22‐HA and MdSUT2.2^S381A^‐Myc/MdCIPK22‐HA calli.
**Figure S14** Expression level of *MdCIPK22* gene and MdCIPK22 proteins in plantlets of three transgenic lines MdCIPK22‐1, MdCIPK22‐3 and MdCIPK22‐5.
**Figure S15** Red fluorescence observation in root of MdCIPK22‐3^shoot^/(MdCIPK22‐3 + anti‐MdSUT2.2)^root^ plant.
**Figure S16** The expression analysis and relative water content was detected in MdCIPK22‐3 and MdCIPK22‐3^shoot^/(MdCIPK22‐3 + anti‐MdSUT2.2)^root^.
**Figure S17** CCB1 and CCB2 domains of SUT2 proteins in different plant species.

**Table S1** Primers used in this study.
