# Supplementary material for: An apple sucrose transporter MdSUT2.2 is a phosphorylation target for protein kinase MdCIPK22 in response to drought
Source: Plant Biotechnol J. 2018 Oct 2;17(3):625–37. doi: 10.1111/pbi.13003 (PMC6381786; doi:10.1111/pbi.13003)

**Supplemental Figure 1.** The phylogenetic tree analysis of MdSUTs and AtSUTs.

**
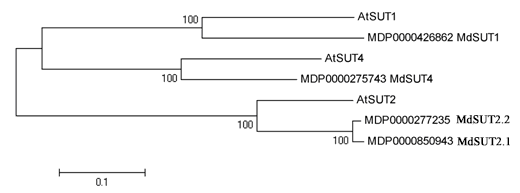
**

**Supplemental Figure 2.** The sequence similarity of AtSUT2, MdSUT2.1 and MdSUT2.2.

**Sequence AtSUT2, MdSUT2.1 and MdSUT2.2 homology respectively was 69.40% and 65.95%**


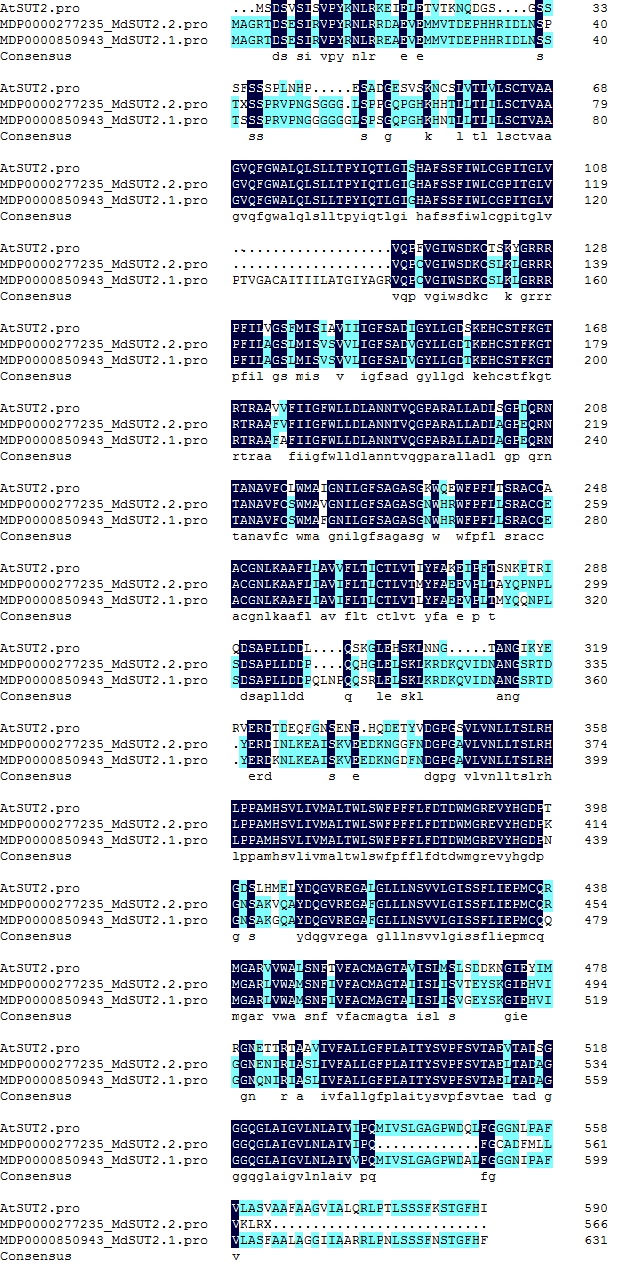


**Supplemental Figure 3.** The expression of MdSUT2.1 and MdSUT2.2 was induced by drought.

**
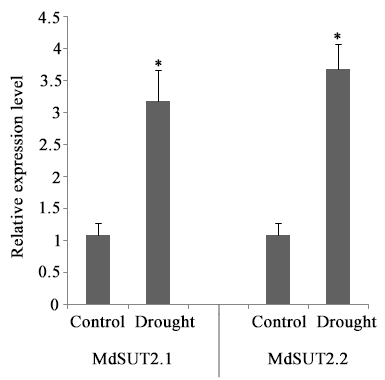
**

**Supplemental Figure 4.** The transmembrane domains of AtSUT2.

**
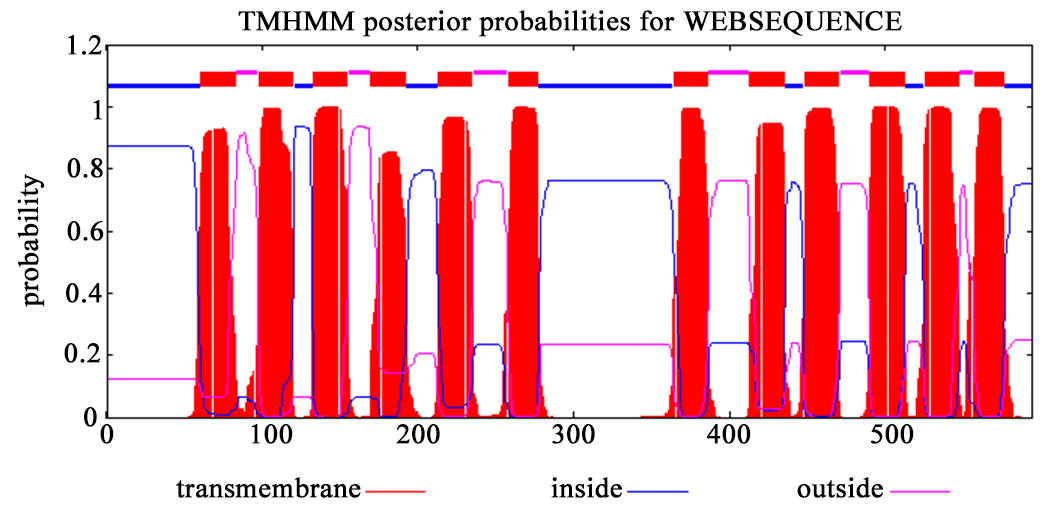
**

**Supplemental Figure 5.** Soluble sugar content and relative water content in three transgenic lines (MdSUT2.2-1, MdSUT2.2-2 and MdSUT2.2-5) and the WT ‘Gala’ control treated with or without drought in plant dry weight. The shoot of WT and three transgenic lines were used to measure the sugar content. Values are the mean of three replicates, and differences with a P-value<0.05 were considered significant. n.s., P>0.01;*P<0.01;**P<0.001.

**
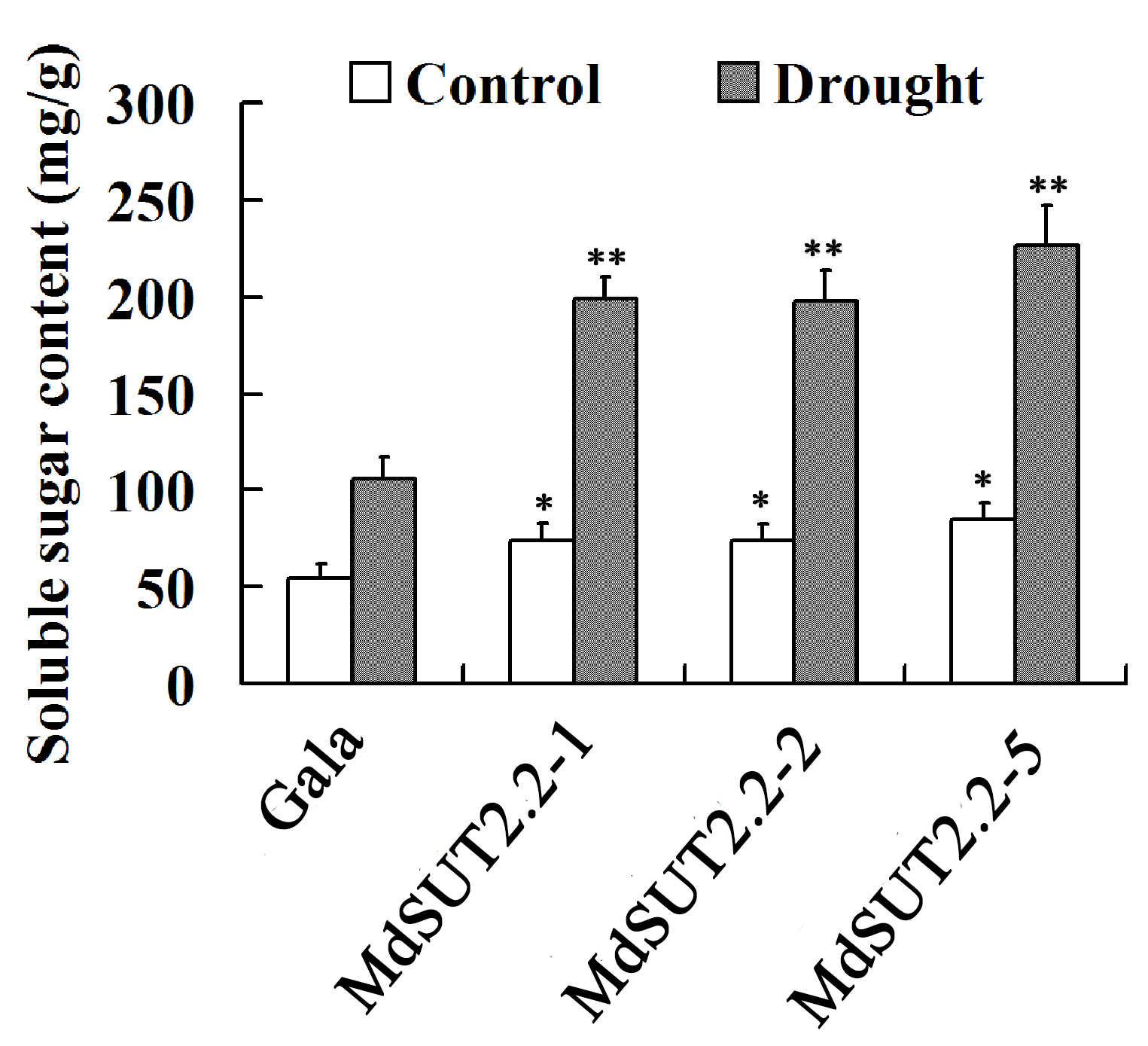

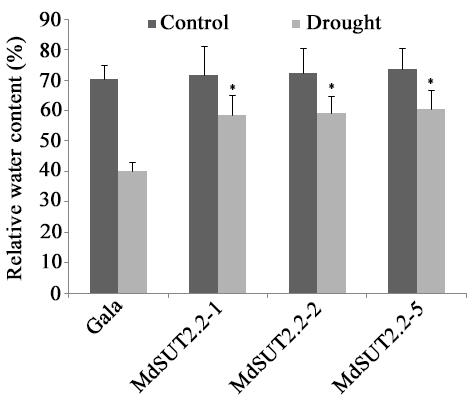
**

**Supplemental Figure 6.** Collision-induced dissociation mass spectrum showed the phosphorylation site was serine (S) at residue 381 (S381) of the MdSUT2.2 protein.

**
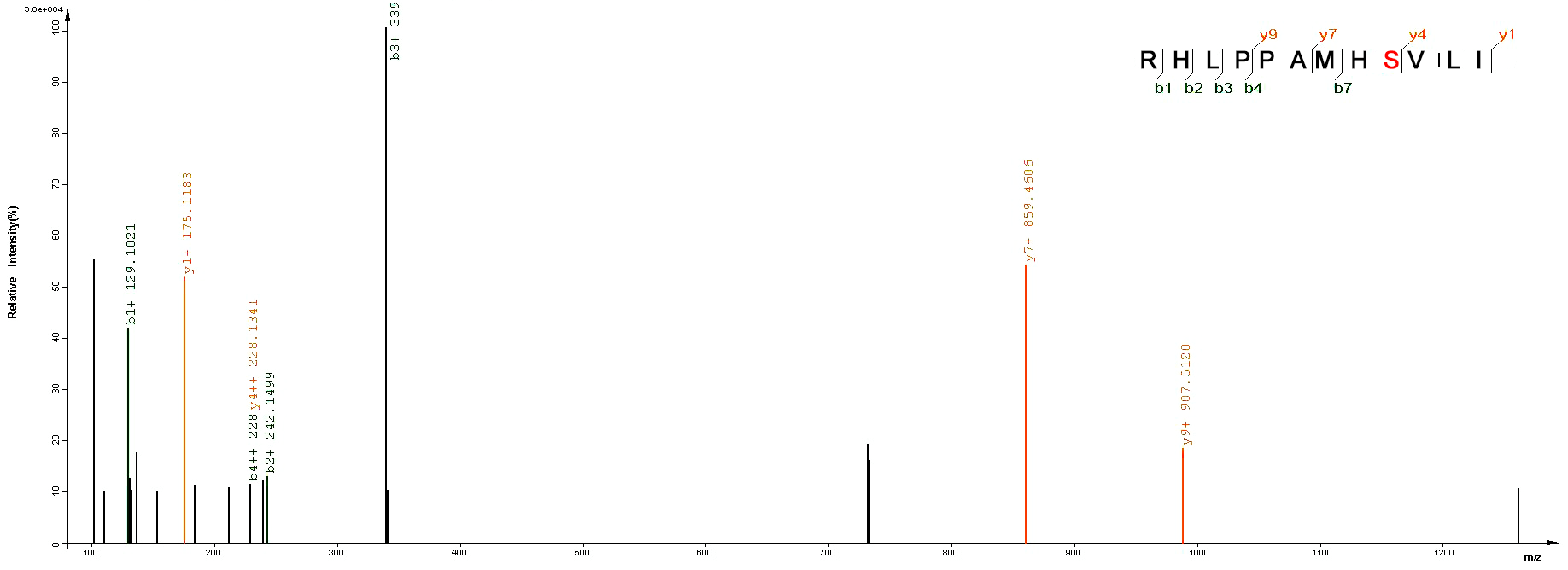
**

**Supplemental Figure 7.** PEG induces phosphorylation for MdSUT2.2 protein in the wild type apple calli.


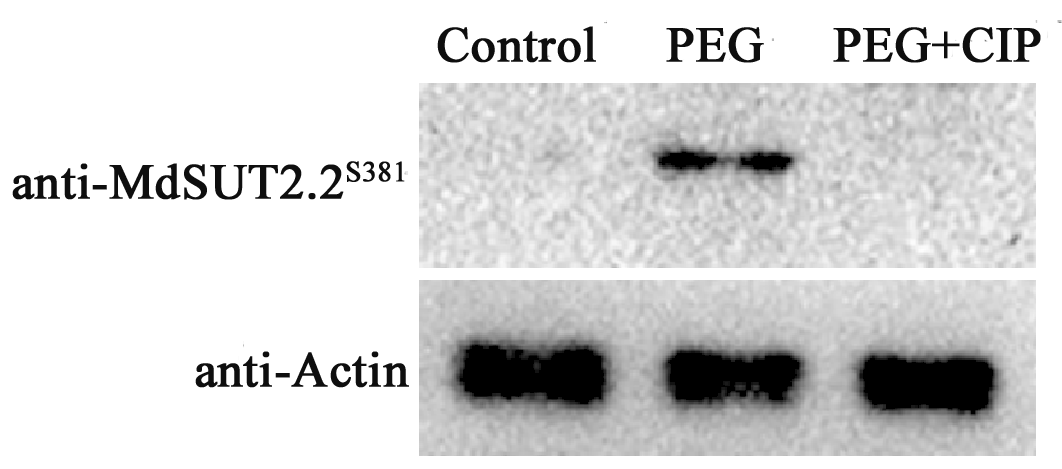


**Supplemental Figure 8.** PEG tolerance of transgenic calli (MdSUT2.2-Myc and MdSUT2.2^S381A^-Myc) and the WT control. (a) The expression of MdSUT2.2 in transgenic calli (MdSUT2.2-Myc and MdSUT2.2^S381A^-Myc); (b) Observation of PEG tolerance of apple calli as indicated; (c-d) soluble sugar (c) and MDA (d) contents in apple calli tested.


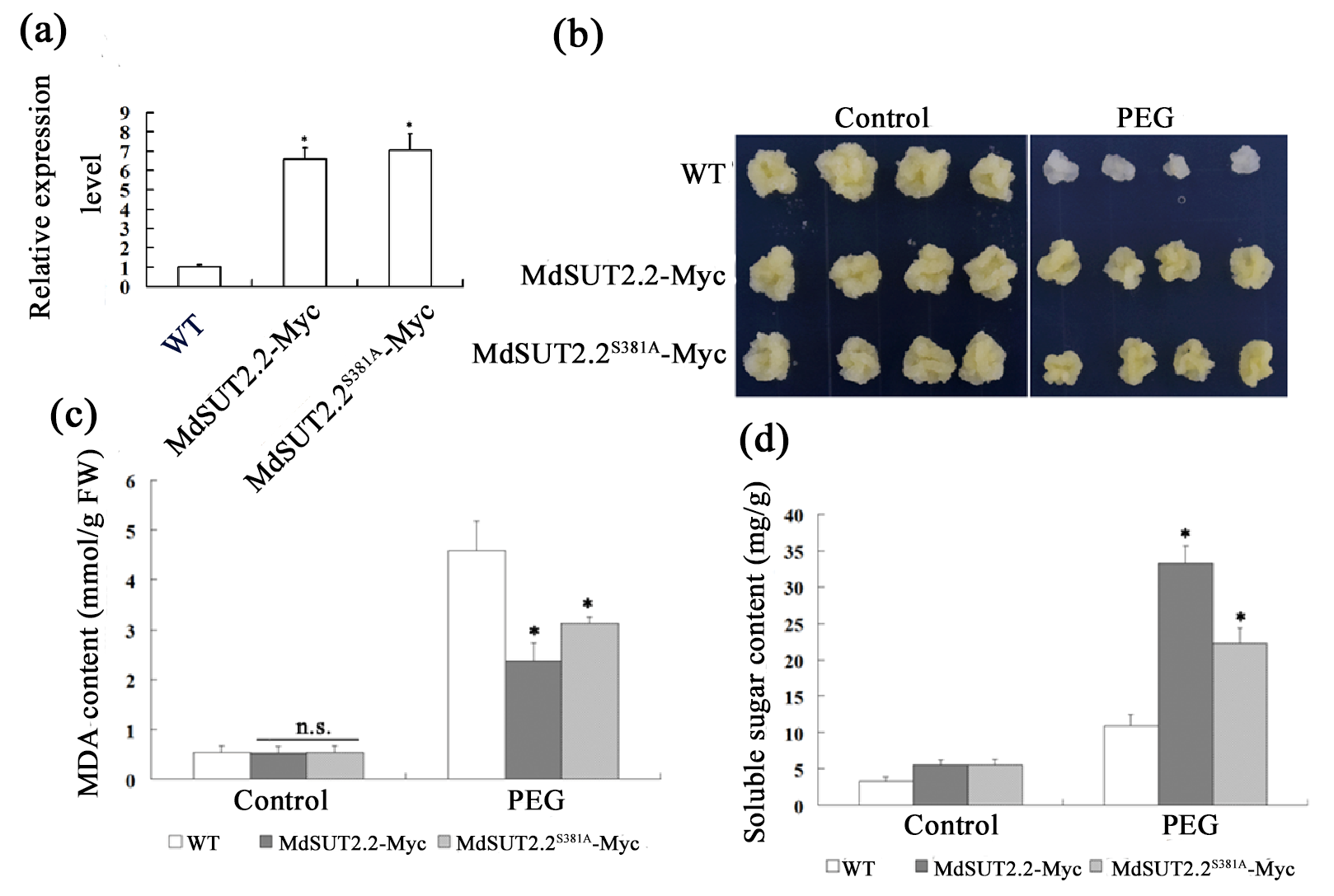


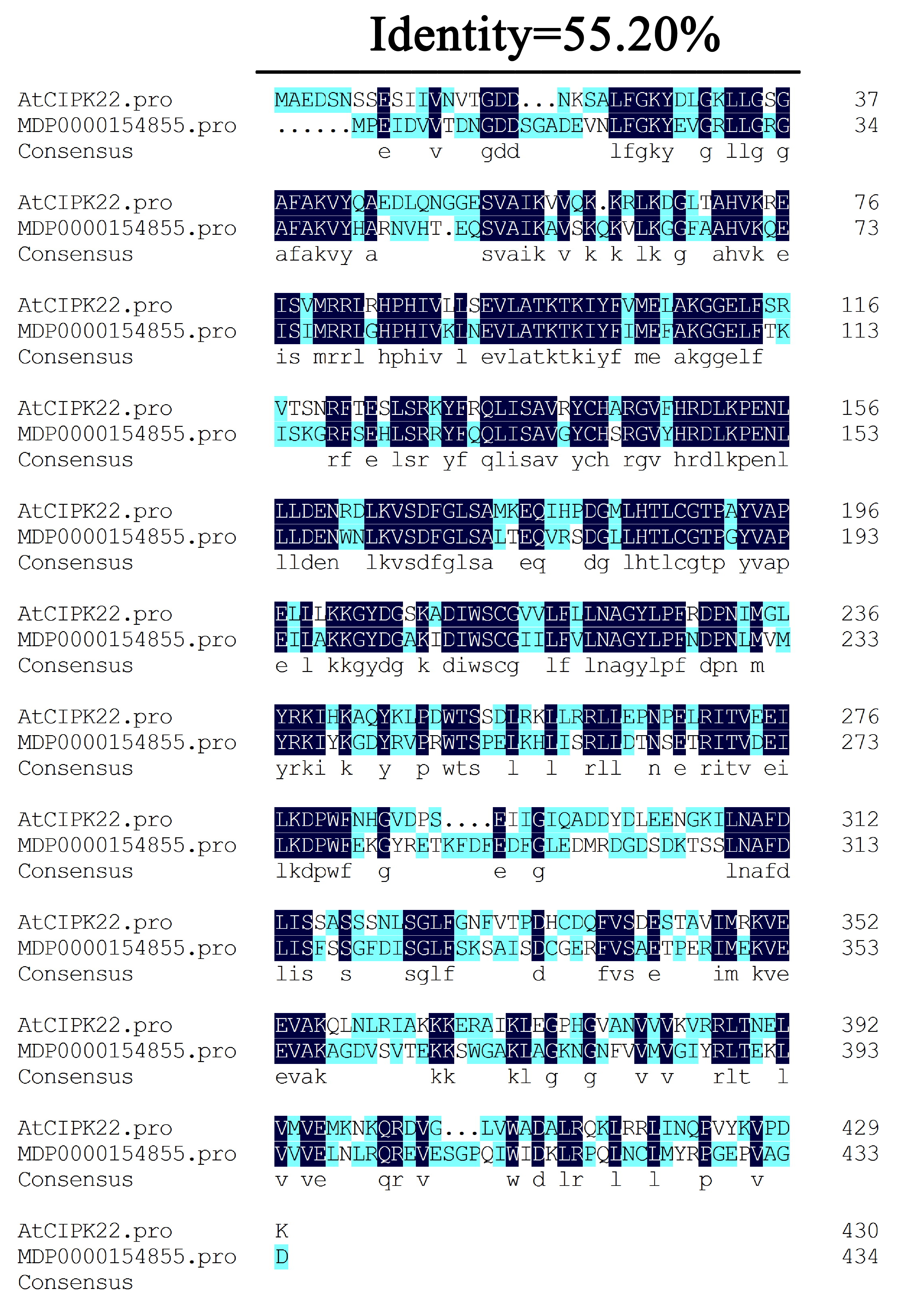
**Supplemental Figure 9.** Amino acid sequence alignment of MDP0000154855 and AtCIPK22

**Supplemental Figure 10.** Functional domain analysis of CIPK proteins in different plant species.


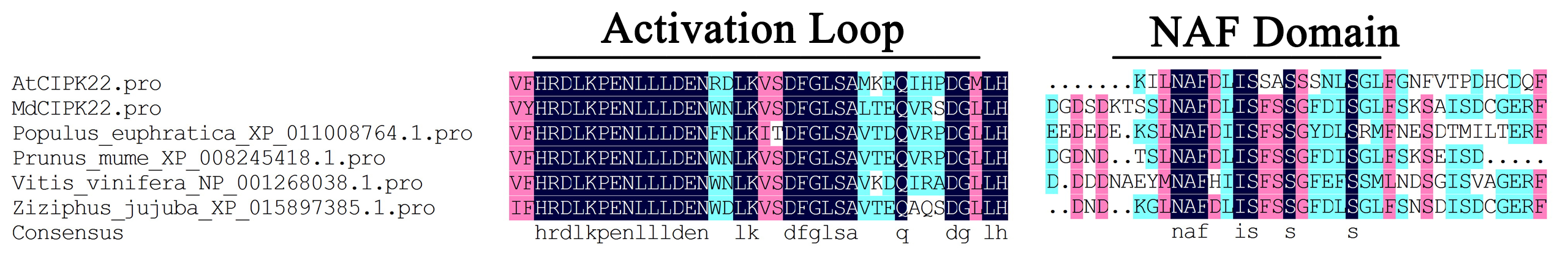


**Supplemental Figure 11.** Pull-Down assay of protein interaction between MdSUT2.2^S381A^-GST and MdCIPK22-His


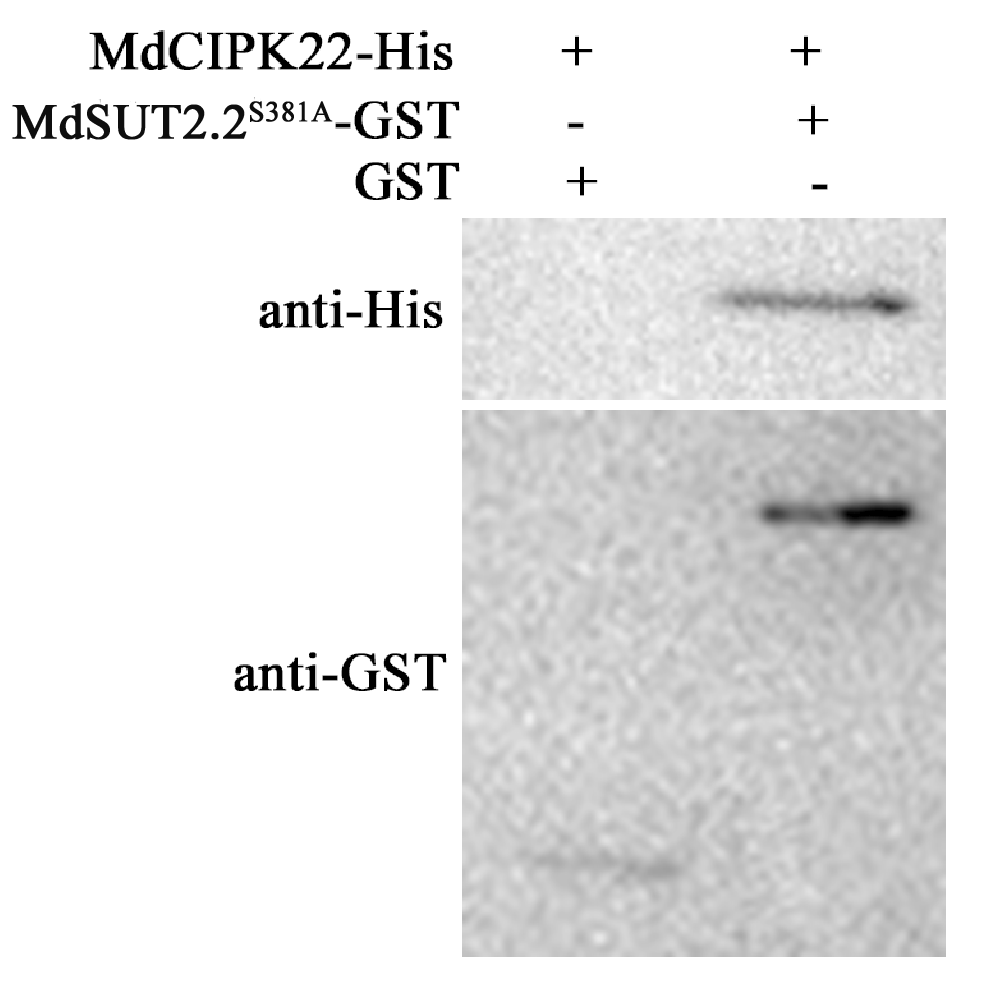


**Supplemental Figure 12.** Expression level of *MdCIPK22* gene in 35S::MdSUT2.2-Myc/MdCIPK22-TRV and 35S::MdSUT2.2-Myc/TRV calli.


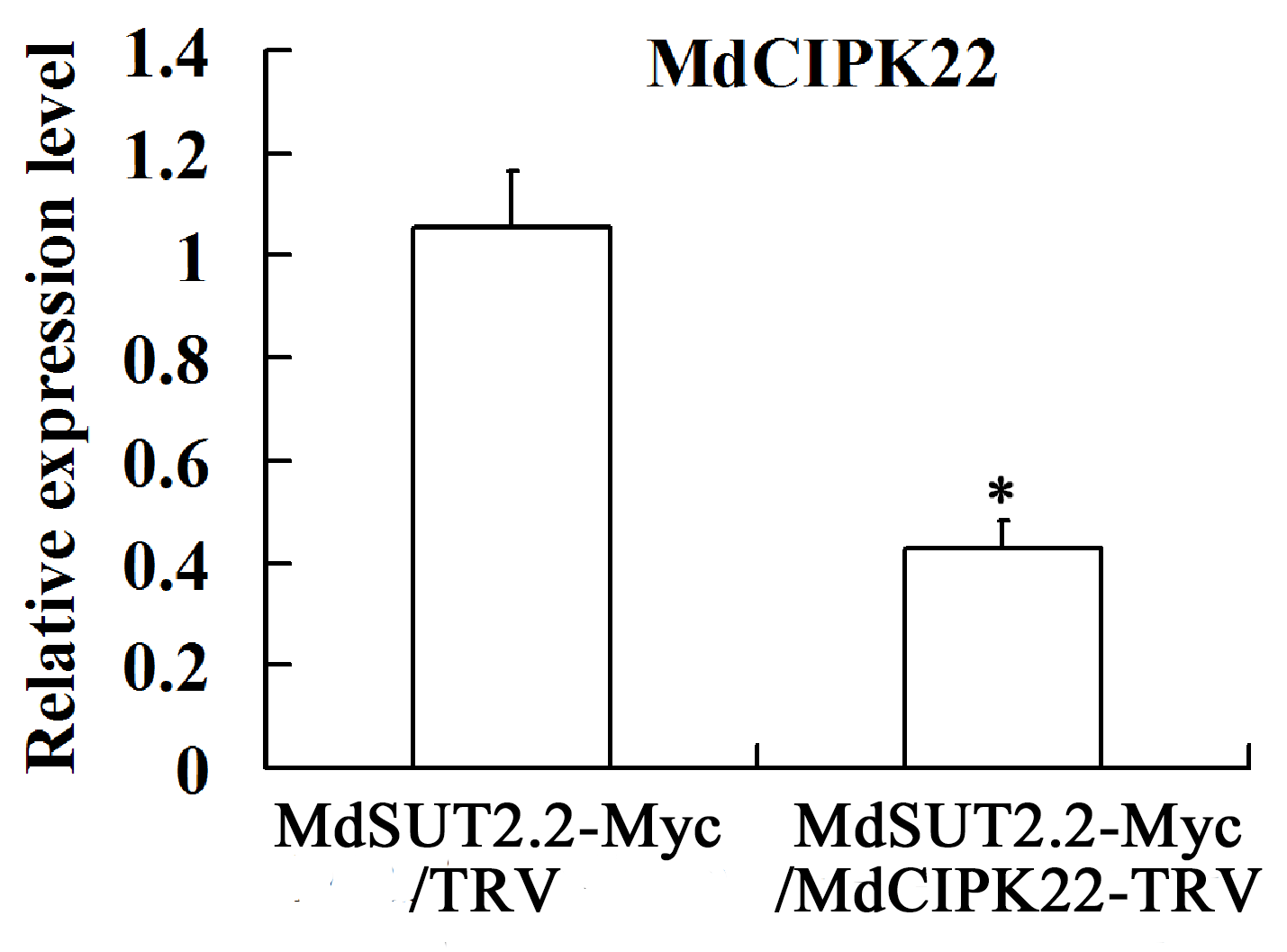


**Supplemental Figure 13.** Expression level of *MdCIPK22* and MdSUT2.2 gene were detected in MdSUT2.2-Myc/MdCIPK22-HA and MdSUT2.2^S381A^-Myc/MdCIPK22-HA calli.


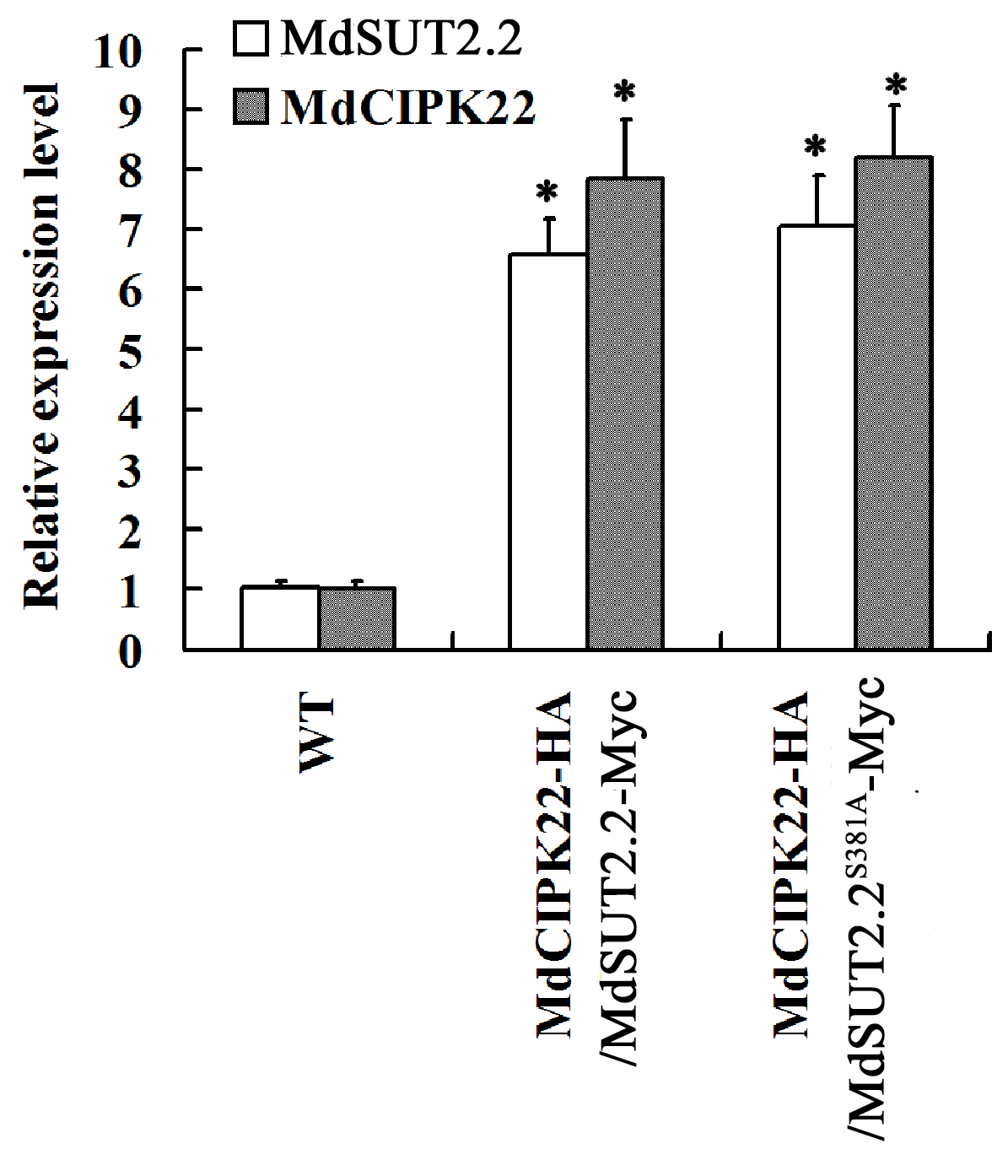


**Supplemental Figure 14.** Expression level of *MdCIPK22* gene and MdCIPK22 proteins in plantlets of three transgenic lines MdCIPK22-1, MdCIPK22-3 and MdCIPK22-5.

**(a)** **(b)**


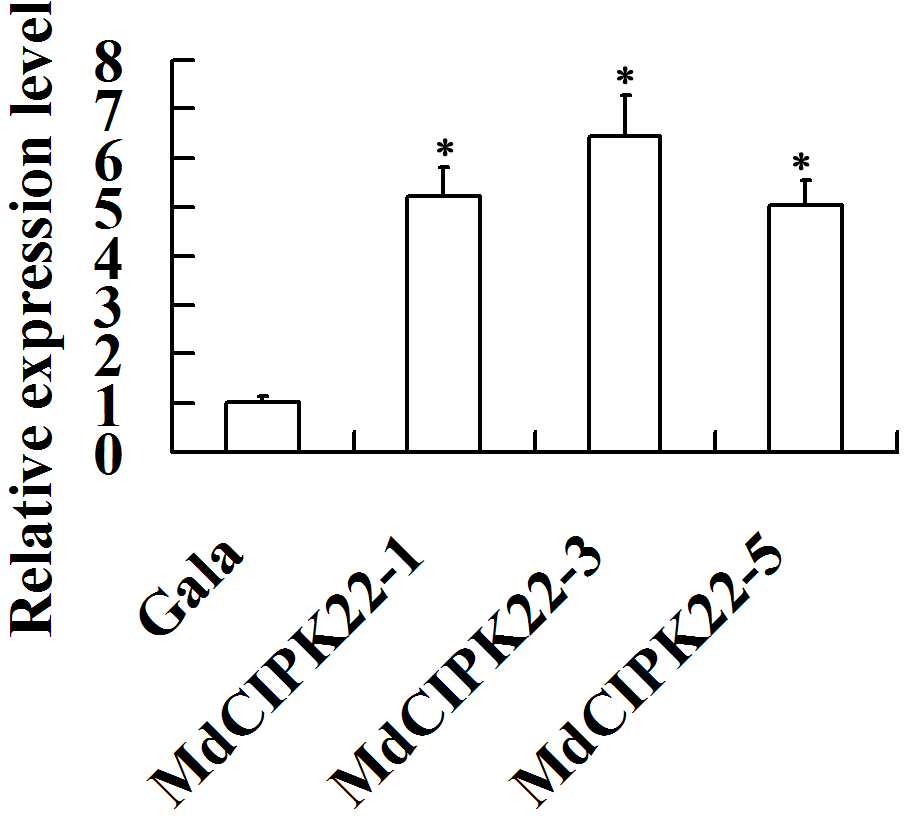


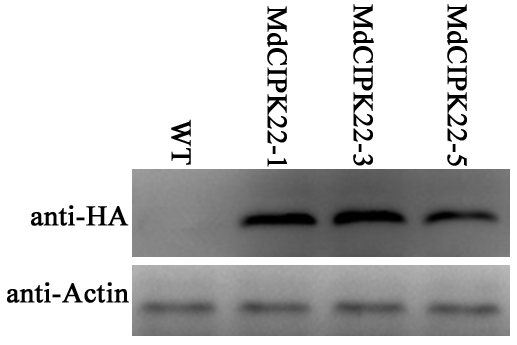


**Supplemental Figure 15.** Red fluorescence observation in root of MdCIPK22-3^shoot^/(MdCIPK22-3+anti-MdSUT2.2)^root^ plant


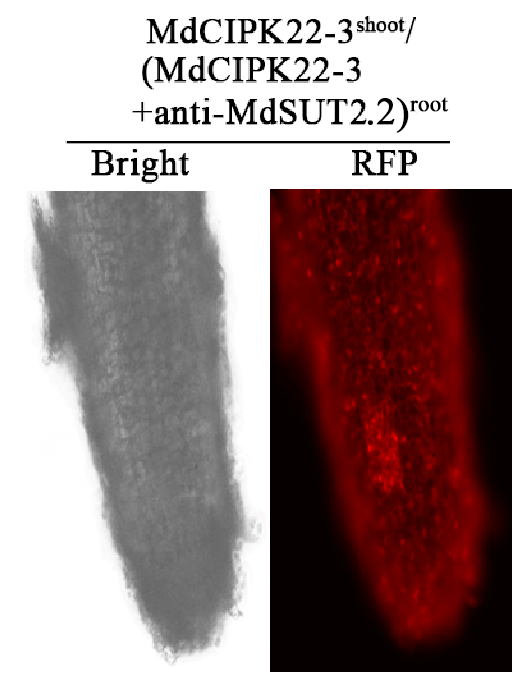


**Supplemental Figure 16.** The expression analysis and relative water content was detected in MdCIPK22-3 and MdCIPK22-3^shoot^/(MdCIPK22-3+anti-MdSUT2.2)^root^. **(a)** Expression level of *MdSUT1*, *MdSUT3* and *MdSUT4* genes in root of MdCIPK22-3^shoot^/(MdCIPK22-3+anti-MdSUT2.2)^root^ plant; **(b)** the relative water content of MdCIPK22-3 and MdCIPK22-3^shoot^/(MdCIPK22-3+anti-MdSUT2.2)^root^ was detected.


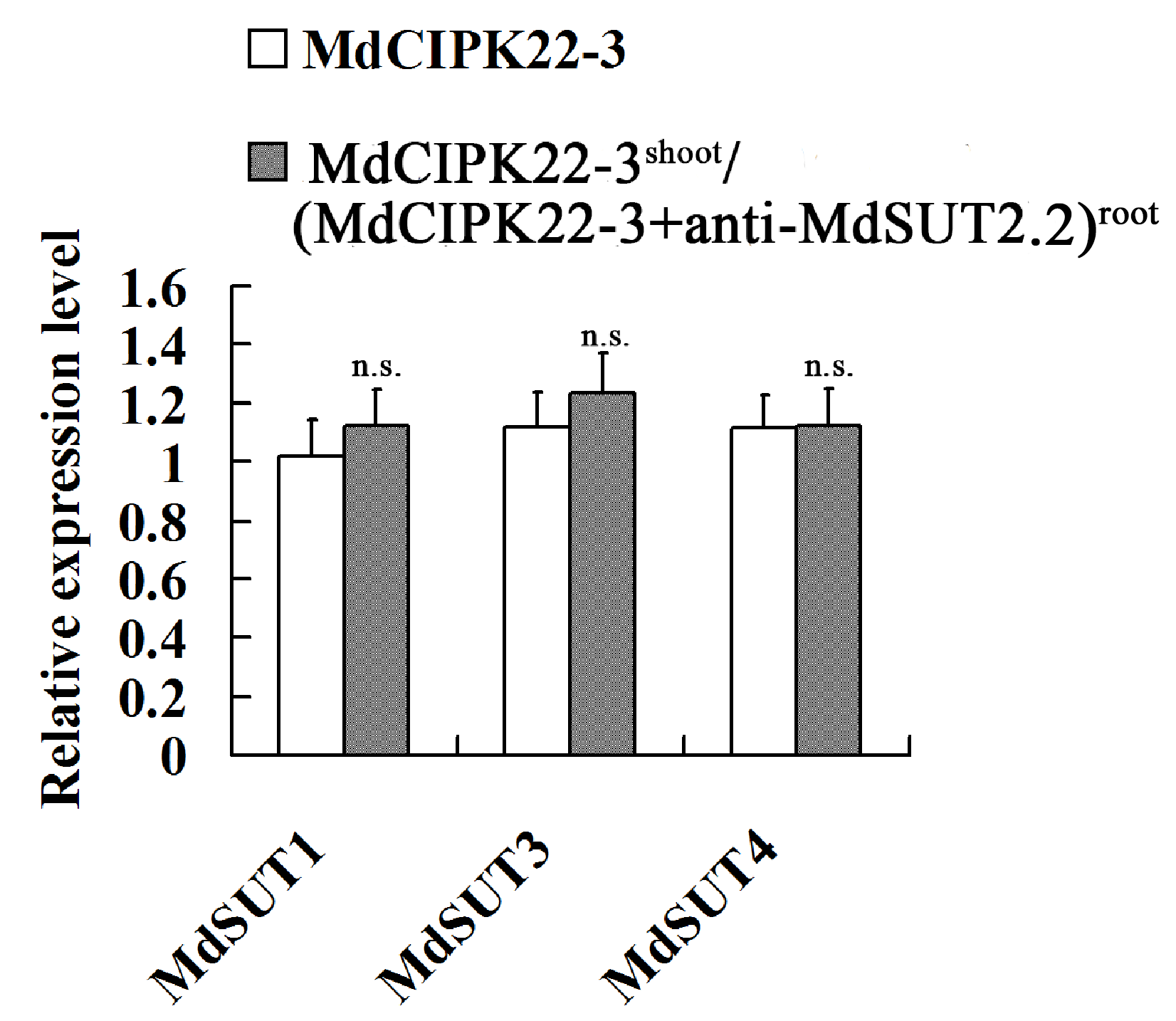
**(a)**

**(b)**


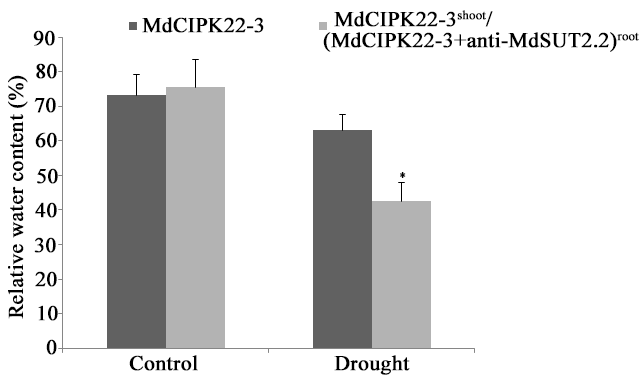


**Supplemental Figure 17.** CCB1 and CCB2 domains of SUT2 proteins in different plant species.


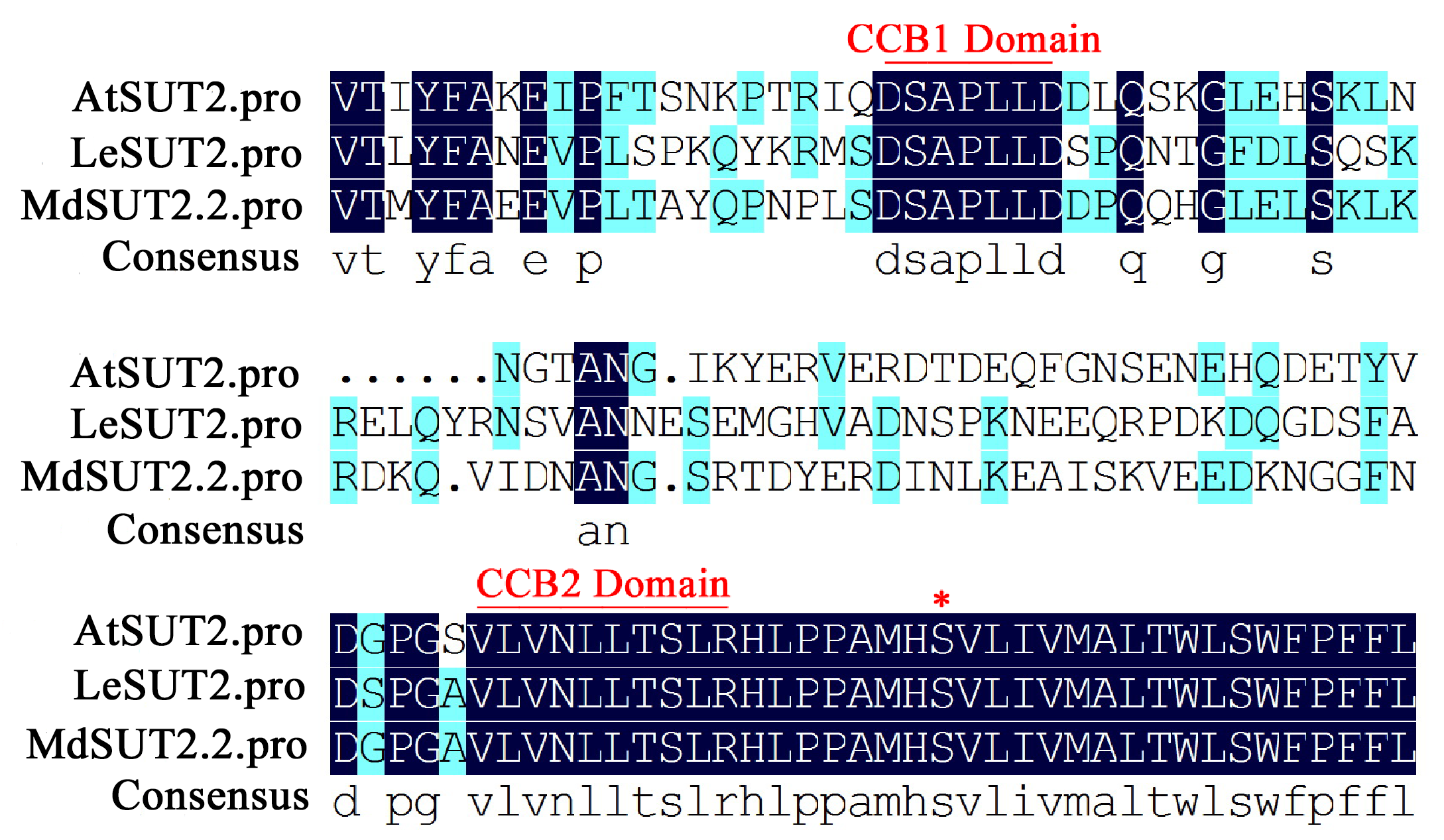

Supplement: Supplementary file 1 — Figure S1 The phylogenetic tree analysis of MdSUTs and AtSUTs. Figure S2 The sequence similarity of AtSUT2, MdSUT2.1 and MdSUT2.2. Figure S3 The expression of MdSUT2.1 and MdSUT2.2 was induced by drought. Figure S4 The transmembrane domains of AtSUT2. Figure S5 Soluble sugar content and relative water content in three transgenic lines (MdSUT2.2‐1, MdSUT2.2‐2 and MdSUT2.2‐5) and the WT ‘Gala’ control treated with or without drought in plant dry weight. Figure S6 Collision‐induced dissociation mass spectrum showed the phosphorylation site was serine (S) at residue 381 (S381) of the MdSUT2.2 protein. Figure S7 PEG induces phosphorylation for MdSUT2.2 protein in the wild type apple calli. Figure S8 PEG tolerance of transgenic calli (MdSUT2.2‐Myc and MdSUT2.2S381A‐Myc) and the WT control. Figure S9 Amino acid sequence alignment of MDP0000154855 and AtCIPK22. Figure S10 Functional domain analysis of CIPK proteins in different plant species. Figure S11 Pull‐Down assay of protein interaction between MdSUT2.2S381A‐GST and MdCIPK22‐His. Figure S12 Expression level of MdCIPK22 gene in 35S::MdSUT2.2‐Myc/MdCIPK22‐TRV and 35S::MdSUT2.2‐Myc/TRV calli. Figure S13 Expression level of MdCIPK22 and MdSUT2.2 gene were detected in MdSUT2.2‐Myc/MdCIPK22‐HA and MdSUT2.2S381A‐Myc/MdCIPK22‐HA calli. Figure S14 Expression level of MdCIPK22 gene and MdCIPK22 proteins in plantlets of three transgenic lines MdCIPK22‐1, MdCIPK22‐3 and MdCIPK22‐5. Figure S15 Red fluorescence observation in root of MdCIPK22‐3shoot/(MdCIPK22‐3 + anti‐MdSUT2.2)root plant. Figure S16 The expression analysis and relative water content was detected in MdCIPK22‐3 and MdCIPK22‐3shoot/(MdCIPK22‐3 + anti‐MdSUT2.2)root. Figure S17 CCB1 and CCB2 domains of SUT2 proteins in different plant species. [file PBI-17-625-s002.doc]
