# Supplementary material for: An apple sucrose transporter MdSUT2.2 is a phosphorylation target for protein kinase MdCIPK22 in response to drought
Source: Plant Biotechnol J. 2018 Oct 2;17(3):625–37. doi: 10.1111/pbi.13003 (PMC6381786; doi:10.1111/pbi.13003)
Supplement: Supplementary file 2 — Table S1 Primers used in this study. [file PBI-17-625-s001.doc]

Supplemental Table 1. Primers used in this study.

| Primer | Sequence (5'to 3') |
| --- | --- |
| MdCIPK22-F | GGAATTGGGATTATTTTCTGG |
| MdCIPK22-R | GGACATGTTTTCTTCCGGTTTC |
| qMdCIPK22-F | CCCCCAAAAGACATCGTAACCAT |
| qMdCIPK22-R | TGTTTTCCGTACCAAGCAAAG |
| MdSUT2.2-F | AGTCGCGGCCATTACTGAGTC |
| MdSUT2.2-R | TCCCCCAAATAAAGCATCCCATG |
| qMdSUT2.2-F | GATCCACCTGCAAAACTCTGCT |
| qMdSUT2.2-R | GGCGACTCGGTTTCACTGACTC |
| 18S -F | CACGGGGAGGTAGTGACAA |
| 18S -R | CCTCCAATGGATCCTCGTTA |
| MdSUT2.2(TRV)-F | CACCATGGCGGGGAGGACGG |
| MdSUT2.2(TRV)-R | CTGTCCCGGCGGCGAGAGGC |
